# Biomimetic Antifungal Materials: Countering the Challenge of Multidrug-Resistant Fungi

**DOI:** 10.3390/biomimetics9070425

**Published:** 2024-07-12

**Authors:** Hazim O. Khalifa, Atef Oreiby, Mohamed A. A. Abdelhamid, Mi-Ran Ki, Seung Pil Pack

**Affiliations:** 1 Department of Veterinary Medicine, College of Agriculture and Veterinary Medicine, United Arab Emirates University, Al Ain P.O. Box 1555, United Arab Emirates; 2 Department of Pharmacology, Faculty of Veterinary Medicine, Kafrelsheikh University, Kafrelsheikh 33516, Egypt; 3 Department of Animal Medicine, Faculty of Veterinary Medicine, Kafrelsheikh University, Kafrelsheikh 33516, Egypt; atef.ibrahim@vet.kfs.edu.eg; 4 Department of Biotechnology and Bioinformatics, Korea University, Sejong-ro 2511, Sejong 30019, Republic of Korea; mohamed42@korea.ac.kr (M.A.A.A.); allheart@korea.ac.kr (M.-R.K.); 5 Department of Botany and Microbiology, Faculty of Science, Minia University, Minia 61519, Egypt; 6 Institute of Industrial Technology, Korea University, Sejong-ro 2511, Sejong 30019, Republic of Korea

**Keywords:** biomimetic, antifungal resistance, antimicrobial peptides, antifungal peptides, alginate-based antifungals, chitosan, chitosan derivatives, nanoparticles, plant-derived polyphenols, probiotic bacteria, graphene-based materials

## Abstract

In light of rising public health threats like antifungal and antimicrobial resistance, alongside the slowdown in new antimicrobial development, biomimetics have shown promise as therapeutic agents. Multidrug-resistant fungi pose significant challenges as they quickly develop resistance, making traditional antifungals less effective. Developing new antifungals is also complicated by the need to target eukaryotic cells without harming the host. This review examines biomimetic antifungal materials that mimic natural biological mechanisms for targeted and efficient action. It covers a range of agents, including antifungal peptides, alginate-based antifungals, chitosan derivatives, nanoparticles, plant-derived polyphenols, and probiotic bacteria. These agents work through mechanisms such as disrupting cell membranes, generating reactive oxygen species, and inhibiting essential fungal processes. Despite their potential, challenges remain in terms of ensuring biocompatibility, optimizing delivery, and overcoming potential resistance. Production scalability and economic viability are also concerns. Future research should enhance the stability and efficacy of these materials, integrate multifunctional approaches, and develop sophisticated delivery systems. Interdisciplinary efforts are needed to understand interactions between these materials, fungal cells, and the host environment. Long-term health and environmental impacts, fungal resistance mechanisms, and standardized testing protocols require further study. In conclusion, while biomimetic antifungal materials represent a revolutionary approach to combating multidrug-resistant fungi, extensive research and development are needed to fully realize their potential.

## 1. Introduction

Fungal infections contribute to a broad spectrum of health issues in humans, ranging from allergic reactions and mucocutaneous infections to severe, life-threatening diseases. These fungal infections typically have a high mortality rate, and achieving a positive clinical outcome necessitates early diagnosis and effective antifungal treatment [1,2,3]. The rising prevalence of these infections is drawing global attention, particularly because they are increasingly common among immunocompromised individuals and patients with severe immunosuppressive conditions [1,2,3,4,5]. By 2023, over 6.5 million people were affected by fungal infections each year, resulting in approximately 3.8 million deaths annually [4,5,6,7]. Of these deaths, 995,000 were attributed to chronic pulmonary aspergillosis, and 214,000 resulted from Pneumocystis pneumonia [8]. Additionally, *Cryptococcal meningitis* accounted for 147,000 deaths, while fungal asthma caused 46,000 fatalities [8]. Furthermore, various other life-threatening fungal infections collectively led to 161,000 deaths annually [8]. The WHO created a fungal priority pathogens list to guide the development of strategies to combat the rising issue of fungal infections and antimicrobial resistance (AFR) [9]. Additionally, systemic fungal infections are frequently diagnosed late, which raises the mortality rate among patients [5]. Furthermore, the burden of fungal infections grew during the COVID-19 pandemic, resulting in higher rates of morbidity and mortality [10,11]. It is important to note that AFR is a natural phenomenon, as fungi are inherently resistant to specific antifungal agents [12]. However, overuse and misuse of these agents have accelerated the development and spread of AFR [6,7]. The issue of AFR is exacerbated by the limited number of available antifungal treatments, which for invasive diseases are confined to a few chemical classes; azoles, echinocandins, polyenes, and flucytosine [6,7]. The development of resistance to any single antifungal class significantly restricts therapeutic options due to the scarcity of treatments. Additionally, multi-drug resistance can eliminate treatment possibilities altogether, leading to devastating effects on patient outcomes.

The development of new antifungal strategies is crucial due to the increasing prevalence of AFR and the limited number of effective antifungal agents currently available. A well-established and successful example of these strategies is antifungal combination therapy [13]. Combination therapies that utilize synergistic interactions between different antifungal agents or between antifungal agents and other types of drugs are being explored to enhance their efficacy and reduce the likelihood of resistance development [13]. For example, when different drugs with unique modes of action are combined, the range of intracellular processes they target expands, making it more challenging for pathogens to develop resistance since they would need mutations in multiple genes [14]. Furthermore, combining therapies can enhance the fungicidal effectiveness of typically fungistatic drugs like azoles, thereby diminishing pathogen populations and lowering the likelihood of resistance emergence [15]. An alternative approach involves reducing fungal virulence, which complements fungicidal and fungistatic agents by preventing the microbe from harming its host [16]. Although targeting non-essential genes is a newer concept, some antibiotics already focus on crucial structures instead of essential proteins, such as daptomycin targeting the Gram-positive cell wall [17]. Targeting virulence factors has several benefits; it expands the range of antifungal targets, reduces the impact on the host’s natural fungal community, and lowers the chance of drug resistance developing [18,19]. Another alternative method for treating fungal infections involves strengthening the host immune system’s ability to combat such infections. There have been endeavors to create preventive immunotherapies and vaccines against fungal pathogens, yielding varying degrees of success. Researchers have concentrated on utilizing a common fungal antigen present in multiple prevalent pathogenic genera, particularly cell wall β-glucans, for the development of vaccines [20]. Other innovative strategies include the use of nanoparticles for targeted drug delivery and the development of vaccines to prevent fungal infections [21]. Recently, nanoparticles have become widely utilized, particularly in targeted drug delivery, tissue engineering scaffolds, and disease diagnostics [22]. A variety of nanoparticle forms, such as carbon nanotubes, nanoliposomes, nanocapsules, and nanofibers, have been extensively used as carriers for drugs and as scaffolds for cellular purposes [21,22].

Another promising approach is the use of biomimetic materials, which imitate natural biological processes and can be engineered to enhance antifungal activity [23]. For example, biomimetic peptides and polymers can be designed to disrupt fungal cell membranes or inhibit essential fungal enzymes [24]. These advances are critical for improving patient outcomes and addressing the growing challenge of AFR. Therefore, this review aims to explore the potential of biomimetic antifungal materials as a promising strategy to address the challenges posed by multidrug-resistant fungi. The review will delve into the principles of biomimicry and how they are applied in the design and development of antifungal materials. It will examine the various biomimetic approaches utilized, such as mimicking natural antimicrobial peptides and structures, to enhance the efficacy of antifungal agents. Furthermore, the review will discuss the advantages and limitations of biomimetic antifungal materials compared to traditional antifungal therapies. Ultimately, the goal is to provide a comprehensive overview of the current state of biomimetic antifungal materials research and their potential applications in combating multidrug-resistant fungal infections.

## 2. Understanding Multidrug-Resistant Fungi and the Challenges for Developing New Antifungals

**Figure 1:**
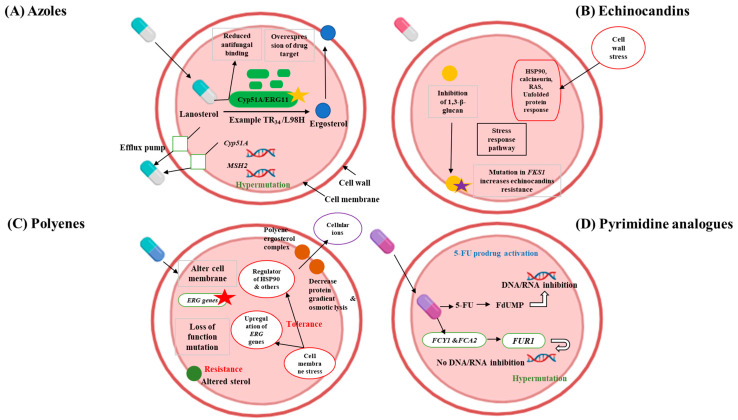
Fungi develop resistance to antifungal drugs through various mechanisms based on the drugs’ mode of action. (**A**) For azole-resistant *Candida*, it is increased drug pumping, while *Aspergillus fumigatus* develops mutations in its *CYP51A* gene. *Cryptococcus neoformans* can become resistant through drug target overexpression and pumping the drug out of the cell. (**B**) Echinocandins work by inhibiting 1,3-β-D-glucan synthase (*FKS1*), and mutations in this gene confer resistance in *Candida* and *Fusarium* species. Echinocandin exposure can also induce cell wall stress by inhibiting β-glucan synthase, which indirectly activates the Ca^2+^/calcineurin or HSP90/mTOR pathways, contributing to drug tolerance. (**C**) Polyene drugs damage fungal cell membranes by binding to ergosterol. Fungi can resist these drugs by acquiring mutations that mess up ergosterol production, especially *Aspergillus* and *Candida* species. In *C. albicans*, losing two copies of the *ERG3* gene leads to resistance and also toleration of the drug by upregulation of other ergosterol-related genes (*ERG5*, *ERG6*, *ERG25*). Additionally, cell membrane stress can affect HSP90 regulators, contributing to drug tolerance. (**D**) Pyrimidine analogues (like 5-flucytosine) block the synthesis of DNA and RNA and can halt the growth of fungi. Resistance to this group can happen due to two main reasons; point mutations in a targeted gene (*FCY1*), especially in *Candida* species, and hypermutation in *Cryptococcus* species. Abbreviations: TR, tandem repeat; 5-FU, 5-fluorouracil; FdUMP, 5-fluorodeoxyuridine monophosphate.

Understanding multidrug-resistant (MDR) fungi is crucial before employing biomimetic approaches to antifungal treatments. MDR fungi like *Candida auris* and *Aspergillus fumigatus* are hard to treat because they resist many antifungal drugs, leading to fewer treatment options and higher death rates. Azole compounds like fluconazole, voriconazole, and posaconazole work by blocking the enzyme cytochrome P450 sterol 14α-demethylase, which converts lanosterol to ergosterol. This enzyme is encoded by *ERG11* in yeast and by *Cyp51* in molds (Figure 1A) [25,26]. The inhibition of sterol 14α-demethylase has a fungistatic effect on yeasts and a fungicidal effect on molds [25,26]. Triazoles are frequently recommended for aspergillosis and are extensively used in the treatment of candidiasis [25]. Epidemiological studies have reported considerable azole resistance in *Candida* and *Aspergillus* species, while resistance in *Cryptococcus* species remains relatively low [26,27,28]. Drug resistance generally emerges through the acquisition of resistance traits and a shift towards species that are naturally less susceptible [29]. Azole resistance in fungi primarily develops through increased drug efflux mechanisms, notably in *Candida* species, and alterations in the sterol biosynthesis pathway due to point mutations and promoter insertions in the *CYP51A* gene in *A. fumigatus* [30]. In *C. neoformans* and other fungi, resistance is frequently attributed to the overexpression of the drug target and efflux pumps, driven by chromosomal aneuploidy and hypermutation. For *Candida* species, resistance mechanisms include the upregulation of drug transporters, overexpression or modification of the drug target, and stress-induced cellular changes [31].

Echinocandin drugs, such as anidulafungin, caspofungin, and micafungin, are lipopeptides that inhibit glucan synthase, a crucial enzyme in cell wall biosynthesis [32]. These drugs are the preferred treatment for various types of candidiasis, with about 60% of candidaemia patients receiving echinocandin therapy [33]. The use of echinocandins has expanded over the past decade [34], increasing the risk of antimicrobial resistance. In *Candida* species, resistance to echinocandins arises exclusively from mutations in the *FKS* genes, which code for the catalytic subunits of glucan synthase (Figure 1B). This resistance is primarily due to amino acid substitutions in two specific hot spot regions of *FKS1* in all *Candida* species and *FKS2* in *Candida glabrata* [7,35]. Moreover, exposure to echinocandins can induce cell wall stress by inhibiting β-glucan synthase. This inhibition indirectly activates downstream pathways such as Ca^2+^/calcineurin and HSP90/mTOR, which play roles in drug tolerance [30]. 

Polyenes, the earliest antifungal drugs, include amphotericin B and nystatin. Amphotericin B, approved in 1957, was originally used to treat severe invasive fungal infections (IFIs). Polyenes function by binding to ergosterol, a sterol unique to fungi, in the plasma membrane. This binding forms concentration-dependent channels that cause cell death by allowing ions and other cellular contents to leak out (Figure 1C). Furthermore, amphotericin B, in its extramembranous aggregate form, is believed to kill cells by extracting ergosterol from lipid bilayers [36]. Historically, amphotericin B was the main treatment for a range of IFIs, including invasive aspergillosis, cryptococcosis, blastomycosis, candidaemia, coccidioidomycosis, histoplasmosis, and mucormycosis. However, due to its renal toxicity, it is now often used as a second-line therapy [5]. Resistance to polyenes occurs through a reduction in ergosterol content in the cell membrane [5,30]. This resistance is typically caused by loss-of-function mutations in genes involved in ergosterol biosynthesis, particularly in *Aspergillus* and *Candida* species. In *C. albicans*, resistance can arise from the double loss of the *ERG3* gene. Moreover, drug tolerance in *C. albicans* is frequently associated with the upregulation of *ERG5*, *ERG6*, and *ERG25*.

Pyrimidine analogues like 5-flucytosine (5-FC), an early antifungal agent, were first synthesized in 1957 as potential anticancer drugs. However, unlike its closely related fluorinated pyrimidine, 5-fluorouracil (5-FU), 5-FC does not possess antineoplastic properties but has antifungal effects instead. Since 1968, 5-FC has been employed to treat human cryptococcosis and candidiasis [37,38,39]. The mode of action of 5-FC is distinct among antifungal agents as it targets DNA, RNA, and protein synthesis (Figure 1D). Being a prodrug, 5-FC needs to be metabolically activated through the pyrimidine salvage pathway, where it acts as a subversive substrate, leading to the formation of toxic nucleotides that disrupt DNA and protein synthesis [37,38]. After entering the fungal cell via membrane permeases (cytosine permease encoded by the *FCY2* gene and its homologs *FCY21* and *FCY22*), 5-FC is transformed into 5-fluoro-uridylate (5-FUMP) through the enzymatic activities of cytosine *deaminase* (encoded by *FCY1*) and uracil phosphoribosyltransferase (UPRT, encoded by the FUR1 gene) [38,39]. Resistance to 5-FC can arise through point mutations in the *FCY1* gene, a common occurrence in *Candida* species. Additionally, hypermutation in *Cryptococcus* species can also lead to resistance against this drug class [30].

The issue of having a limited number of antifungal drugs would be less concerning if the outcomes for invasive fungal infections were generally positive. Unfortunately, this is often not the case. For example, the 90-day survival rate after a candidemia diagnosis varies between 55% and 70%, depending on the patient’s underlying health conditions and the specific species causing the infection [40]. One major challenge in evaluating fungal infection outcomes is differentiating mortality caused by the infection itself from mortality resulting from other comorbidities. The situation is even more critical for aspergillosis, where prognoses remain poor despite the use of voriconazole [41]. Furthermore, developing antifungal drugs presents a unique challenge compared to antibacterial agents due to the close evolutionary relationship between fungi and humans. This similarity is highlighted by the use of *Saccharomyces cerevisiae* as a model eukaryotic organism, which demonstrates that many fundamental biochemical and cellular processes are conserved across fungi and humans. Consequently, numerous compounds that are toxic to yeast are also harmful to humans [42]. Therefore, it is unsurprising that the primary classes of antifungal drugs target structures specific to fungi. Beyond the scientific difficulties in discovering new lead compounds, evaluating new antifungal agents is further complicated by clinical trial design challenges [43]. These issues are exacerbated by broader scientific, economic, and regulatory obstacles that hinder the development of anti-infectives overall [44]. As a result, the progress of antifungal drug development lags considerably behind other therapeutic fields. Bridging this gap will require innovative approaches. One promising strategy could be the development of biomimetic antifungal materials.

## 3. Biomimetic Materials: An Overview

Over the past decade, biomimicry—the practice of imitating natural biological processes—has become a prominent topic in scientific research, particularly within the fields of engineering, biology, and materials science. These natural designs have particularly inspired materials scientists working on ‘smart’ biostructures. Prominent examples include the honeycomb of beehives, the durability of spider silk, the intricate patterns of butterfly wings, the robustness of mollusk shells, the adhesion of gecko feet, the water repellency of lotus leaves, the texture of shark skin, the mechanics of bird flight, the strength of mussel byssus, and the optical properties of venus’ flower basket and brittle star [45,46,47,48,49]. These natural phenomena surpass conventional engineering capabilities, prompting scientists to develop bioinspired materials that replicate these unique characteristics. These innovations, known as biomimetic materials, are created by emulating the designs found in nature. This concept is deeply ingrained in human culture, reflecting our inherent tendency to replicate aspects of the natural world [45]. The term “biomimetics” derives from the Greek words “bios” (meaning “life”) and “mimesis” (meaning “to imitate”) [46]. A notable example of biomimicry is found in the development of medical materials. Biomimetic medical materials are designed to be biocompatible and biodegradable by closely studying natural models and replicating their structures and processes [47]. These materials are used in various medical applications, including biosensing, tissue engineering, regenerative medicine, biosignals, and drug/protein delivery. Additionally, biomimetic biomaterials have significant applications in biomedical fields, particularly in tissue engineering and drug delivery systems [48]. Furthermore, biomimicry has emerged as a promising approach to combating antibiotic-resistant bacteria across diverse domains [49]. Common strategies to tackle antibiotic resistance, which is reported as a major worldwide threat [50,51], encompass molecular-based techniques, biopolymers, microorganisms, and materials originating from naturally antimicrobial sources like platelet-rich plasma (PRP) [49]. Molecular-based methods center on imbuing materials with antimicrobial properties, employing agents such as antimicrobial peptides (AMPs) and nanometal particles like gold, silver, and zinc oxide [52]. Biopolymer strategies entail modifying polymeric materials to possess antimicrobial characteristics, achieved through techniques such as structural alterations, incorporation of leukocyte migration-inducing agents, and the addition of antimicrobial substances [53]. The utilization of naturally derived antimicrobial agents involves harnessing biologically sourced antimicrobials, such as PRP and materials infused with essential oils [54,55]. Furthermore, bacteriophages stand out as an effective strategy for combating bacterial infections due to their remarkable ability to specifically target and eradicate bacteria [56]. Regarding antifungal resistance (AFR), natural products with potent antifungal activity and minimal toxicity to eukaryotic cells represent a promising strategy to combat the spread of resistant fungi [57]. One such example is the AMPs produced by various organisms [58,59,60]. Alongside these natural compounds, other strategies rooted in biomimicry have been developed, which will be comprehensively discussed in this review.

## 4. Types of Biomimetic Antifungal Agents and Strategies

### 4.1. Antifungal Peptides

**Figure 2:**
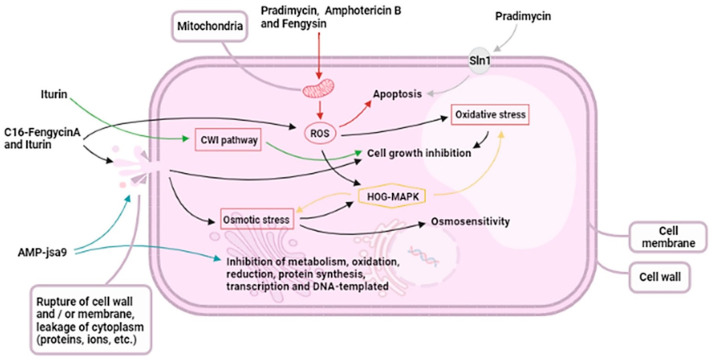
The mechanism of action of some antifungal peptides. Reproduced with permission from [24], copyright 2021 MDPI (CC BY 4.0 DEED).

**Figure 3:**
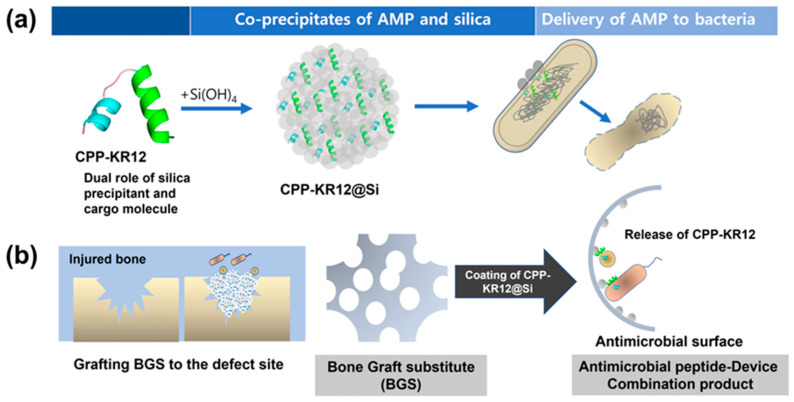
Self-encapsulation of antimicrobial peptides (AMPs) within a silica matrix is achieved through CPP-KR12-mediated silica deposition (**a**), and the creation of a combination product that integrates an antimicrobial peptide with a device (**b**). Reproduced with permission from [62], copyright 2023 MDPI (CC BY 4.0 DEED).

Antifungal peptides, either natural or their analogue synthetic peptides, represent a vital class of biomimetic materials with promising applications. These peptides, including defensins, histatins, and cecropins, exhibit potent antifungal activity by disrupting fungal cell membranes, leading to cell death [61]. Their low toxicity towards mammalian cells makes them particularly attractive for treating multidrug-resistant fungal infections. Additionally, the availability of various antimicrobial peptide databases serves as a valuable resource for researchers, showcasing a wide range of antifungal mechanisms (Figure 2) [24]. Recognizing the limitations of natural antimicrobial peptides, researchers can explore chemical modification to enhance stability and target specificity, potentially leading to improved therapeutics against multidrug-resistant pathogens (Figure 3) [62]. This section will focus on significant antimicrobial peptides, highlighting their antifungal effects.

#### 4.1.1. Cecropin Peptides

Cecropin peptides were initially and primarily identified in insects, hence they are often referred to as insect cecropin peptides. However, they can also be sourced from amphibians, mammals, or synthesized artificially. These peptides disrupt the permeability of fungal cell membranes, induce destructive changes in the cell wall, and cause mitochondrial membrane alterations in *C. albicans* [63]. Nevertheless, not all cecropin peptides exhibit antifungal properties. Many insect-derived cecropin peptides have demonstrated significant antifungal effects, such as AngCec A from *Anopheles gambiae* [64], AeaeCec 1 from *Aedes aegypti* [65], Cec TY1 from *Tabanus yao* [66], Cec A and Cec B from *Drosophila melanogaster* [67], stomoxyn from *Stomoxys calcitrans* [68], Cec A and Cec B from *Hyalophora cecropia* [69], Cec A from *Bombyx mori* [70], papiliocin from *Papilio xuthus* [71], and hinnavin I and hinnavin II from Artogeia rapa [72]. Additionally, some synthetic cecropin peptides have shown antifungal activity, such as D-Cec B, a modified form of *Antheraea pernyi* Cec B [73]; CAMs, a hybrid of *Hyalophora cecropia* Cec A and *Apis mellifera* Mellitin [74], and CA-Mas, a hybrid of *Hyalophora cecropia* Cec A and *Xenopus laevis* Magainin 2 [75].

#### 4.1.2. Defensins and Defensin-like Peptides

Defensins are a diverse group of antimicrobial peptides rich in cysteine, produced by eukaryotes, known for their significant antifungal activity [76]. These peptides are widely distributed and are found in most eukaryotes [77], and defensin-like peptides have also been identified in prokaryotes, such as the bacterium *Anaeromyxobacter dehalogenans* [78]. Defensins share common structural features with various chemical modifications, typically being small molecules composed of up to 50 amino acids [79]. Even a single amino acid substitution can alter their activity spectrum [80]. They are effective and safe antifungals at low concentrations ranging from 0.1 to 10 μM [81]. Silva et al. (2014) state that vertebrate defensins are categorized into α-, β-, and θ-types, while defensin-like peptides from invertebrates, fungi, and plants are similar in structure to vertebrate β-defensins, indicating a shared ancestor for these peptides [76]. Defensins work by disrupting cell walls, cell membranes, and mitochondrial membranes, and by interfering with vital cellular processes, causing apoptosis and fungal cell damage [75]. However, their amphiphilic, cationic, and protease-sensitive nature gives them a short serum half-life, making them unsuitable for systemic use [82]. This issue might be addressed with specific chemical modifications [83].

#### 4.1.3. Cathelicidins

Cathelicidins are antimicrobial peptides primarily produced by mammals, including humans, cattle, monkeys, mice, rats, rabbits, guinea pigs, pigs, sheep, goats, horses, and dogs [84,85,86,87]. They have also been identified in some non-mammalian species, such as snakes [88], certain fish [89], and chickens [90]. Cathelicidins exert their antifungal effects on various fungal species, including *Aspergillus*, *Candida*, *Colletotrichum*, *Fusarium*, *Malassezia*, *Pythium*, and *Trichophyton*. They do this by damaging the cell wall, altering the cell membrane’s permeability, inducing oxidative stress, disrupting the endoplasmic reticulum, creating autophagy-like structures, negatively affecting fungal genetic material, and inhibiting other cellular functions [91]. According to Tomasinsig and Zanetti (2005), cathelicidins consist of two functional domains; a highly conserved N-terminal and a highly variable C-terminal [86]. Van Eijk et al. (2020) tested the antifungal activity of synthesized cathelicidin-like peptides, demonstrating their efficacy against medically significant fungi, including azole-resistant *Aspergillus* fumigatus, even at low concentrations [92].

#### 4.1.4. Dermaseptins

Dermaseptins are a diverse group of antimicrobial peptides, each with a nearly unique amino acid sequence, produced as skin secretions by amphibians such as frogs [93]. Most dermaseptins have a short amino acid chain, typically comprising 21 to 34 residues [94]. They exert their effects by inducing cytotoxicity, disrupting membrane lipids, and triggering apoptosis. This involves the production of reactive oxygen species and DNA fragmentation [95]. Additionally, Belmadani et al. (2018) discovered that some dermaseptins can alter gene expression, such as dermaseptin-S1 which modulates the hyphal wall protein 1 in *C. albicans* [96].

### 4.2. Alginate-Based Antifungals

**Figure 4:**
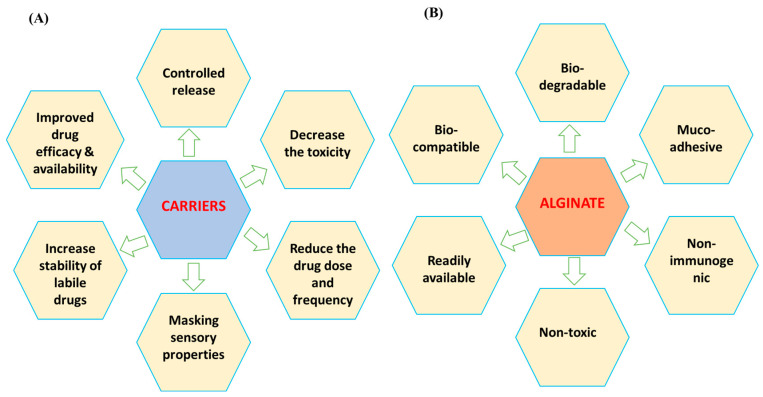
Diagram explaining the advantages of using carriers to deliver drugs (**A**) and highlighting the specific benefits of using alginate, a natural polymer, for this purpose (**B**). Adapted from [98].

Alginates are polysaccharide polymers, derived from various species of sea brown algae [97], which have numerous other medical applications. They are often combined with nano- or microparticles to form hydrogels, tablets, or films for antifungal delivery, offering advantages such as being non-toxic, non-immunogenic, and cost-effective (Figure 4) [98]. Among the different types of alginates, sodium alginate polymer is particularly notable for its unique properties. It serves as a carrier base for antifungal nanocomposite hydrogels, enhancing their synergistic effects. The hydroxyl and carboxylate groups in alginate interact strongly with metal nanoparticles, creating mechanically stable, non-aggregatable, and highly reactive antifungal nanoparticles [99]. Furthermore, alginate-based hydrogels are widely recognized for their biocompatibility, biodegradability, and capacity to mimic the extracellular matrix [100]. These hydrogels can be engineered to incorporate antifungal agents, enhancing their therapeutic efficacy [98]. For example, alginate hydrogels loaded with amphotericin B have demonstrated significant antifungal activity against *Candida* species, a common pathogen encountered in clinical settings [98]. Additionally, these hydrogels can be tailored to control the release rate of antifungal drugs, thereby reducing toxicity and improving patient outcomes [101]. Other biomimetic hydrogels, such as those derived from collagen or gelatin, also exhibit potential in antifungal therapy. These hydrogels can provide a scaffold for tissue regeneration while delivering antifungal agents locally, minimizing the systemic side effects [102]. Furthermore, alginate–copper oxide nanocomposites were tested against *A. niger* and demonstrated effective antifungal responses [103]. Similarly, sodium alginate–silver nanoparticles have been evaluated as antifungals in agriculture, showing efficacy by altering cell membrane permeability, inhibiting protein synthesis, and negatively affecting DNA replication, without harming the plant seeds [104]. Additionally, oxidized alginate-forming aldehyde alginate–silver nanoparticles were studied for their antifungal effects against various fungi, including phytopathogenic fungi, and were found to negatively impact fungal hyphae [104]. Other effective formats of alginates, including zinc alginate fibers [105] and calcium alginate microspheres incorporating essential oils [106], were also reported to have antifungal activities.

### 4.3. Chitosan and Chitosan Derivatives

**Figure 5:**
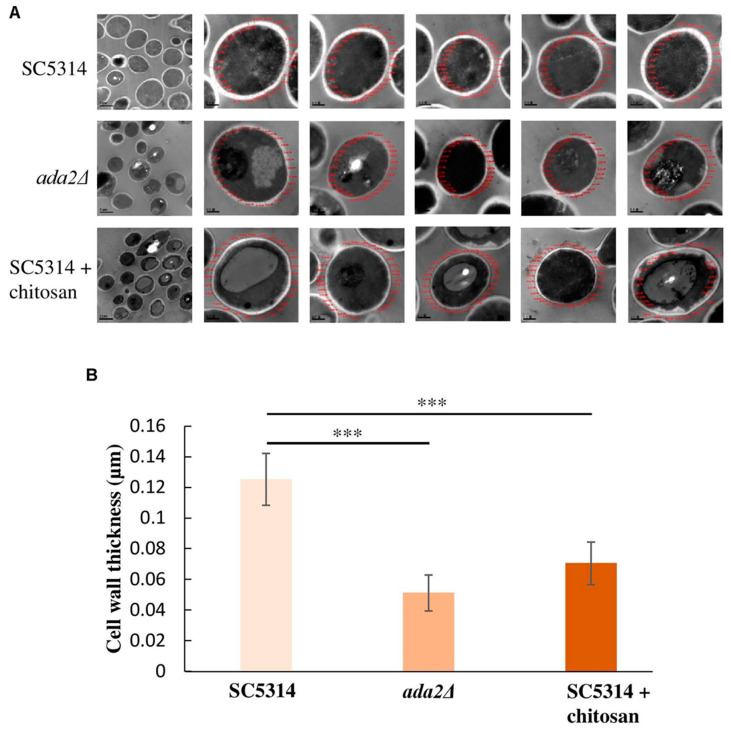
Chitosan-mediated disruption of *Candida albicans* cell wall integrity. Treatment with chitosan significantly reduced cell wall thickness in both a mutant strain lacking a specific enzyme (*ada2*Δ) and the wild strain (SC5314). (**A**) Researchers measured the cell wall thickness under a powerful microscope at high magnification on multiple randomly chosen cells. (**B**) They found that the cell wall of the mutant strain was thinner than in the wild strain. Importantly, chitosan treatment caused a similar decrease in cell wall thickness in the regular strain to that seen in the mutant. This suggests that chitosan might disrupt the cell wall structure of *C. albicans*. *** *p* < 0.001 compared with the value for untreated wild-type SC5314 cells. Reproduced with permission from [115], copyright 2019 Frontiers (CC BY 4.0 DEED).

**Figure 6:**
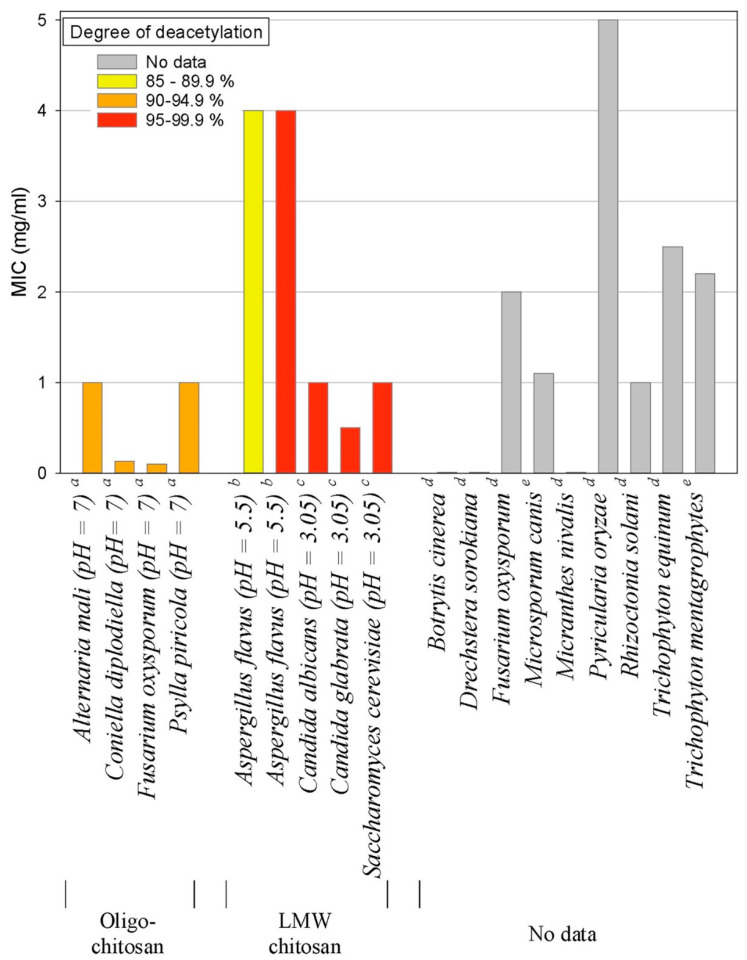
The effect of chitosan on different fungal species. The minimum inhibitory concentration of native chitosan against various fungi (references in the figure: (*a*) [126], (*b*) [127], (*c*) [128], (*d*) [129], (*e*) [130]). Reproduced with permission (license number 5811471104294) from [113], copyright 2017 Elsevier B.V.

Chitosan is a non-toxic and cost-effective biopolymer polysaccharide derived from the hard shell chitin of crustaceans like crabs and shrimp [106], fungi [107], and insects [108]. The ratio of its two constituent monomers and its molecular weight influence chitosan’s water solubility and antifungal properties [109]. Studies have shown that chistosan with a low molecular weight is more effective against mycelia, while chistosan with a high molecular weight is better at inhibiting spore formation [110,111]. However, some research, such as that by Rahman et al. (2015), indicates that antifungal activity decreases with lower molecular weight [112]. Thus, chitosan’s activity is influenced by multiple complex factors that require careful investigation. Other factors affecting its antimicrobial effects include the degree of deacetylation, pH, temperature, and salt presence [113]. Chitosan’s antimicrobial effects increase at high temperatures and low pH, particularly for long-chain chitosan [114]. It disrupts fungal cell membranes by inducing permeability changes and binding to the organism’s DNA [115,116]. Verlee et al. (2017) explained that these permeability changes result from the electrostatic interaction between positively charged chitosan and negatively charged cell membrane phospholipids [113]. At molecular levels, in *C. albicans*, Shih et al. 2019 confirmed that chitosan induces its antifungal effect via inhibition of the Spt-Ada-Gcn5-acetyltransferase (SAGA) complex component expression with subsequent alteration of *Candida* cell surface integrity (Figure 5) [115]. The sensitivity of fungi to chitosan is also influenced by the nature of the cell membrane’s fatty acids, with higher unsaturated fatty acid content linked to increased chitosan activity [117]. Chitosan-induced permeability changes lead to cell membrane destruction, leakage of cellular contents, and chitosan entering the cell to affect genetic material and protein synthesis pathways, ultimately causing cell death [116,118]. This explains why some fungi are resistant to chitosan, as its effectiveness depends on factors such as cell membrane fluidity, molecular weight, and the source of chitosan [113]. Chitosan has been proven effective against many fungal species, including *Botrytis cinerea*, *Fusarium oxysporum*, *Drechstera sorokiana*, *Micronectriella nivalis*, *Piricularia oryzae*, *Rhizoctonia solani*, *Trichophyton equinum*, *F. solani*, *Colletotrichum lindemuthianum*, *Cylindrocladium floridanum*, *A. flavus*, *Botryosphaeria* species, *Candida* species, *T. rubrum*, *A. fumigatus*, and others (Figure 6) [113,119,120,121,122,123,124,125,126,127,128,129,130]. However, some fungi, such as *Saccharomyces cerevisiae*, *Pochonia chlamydosporia*, and *Beauveria bassiana*, are resistant to chitosan [117,131]. 

Various chitosan derivatives have been developed to enhance its properties and antimicrobial effects, which are generally lower than those of commercial antifungals [113]. Phosphorylation improves solubility, quaternization enhances both solubility and antimicrobial activity, and N-alkyl and N-benzyl chitosan derivatives, among other modifications and substitutions, exhibit better antimicrobial effects [113]. Additionally, the degree and type of substitution are crucial factors influencing the antifungal effects of chitosan derivatives. Chitosan nanoparticles have been prepared and shown to be effective antifungals and inhibitors of sporulation, even at very low concentrations [132]. Studies have also evaluated chitosan in film and liquid forms, confirming that its antifungal activity varies depending on the type of fungus being treated [133].

### 4.4. Nanoparticles

Nanoparticles are emerging as promising agents for combating fungal infections due to their distinct characteristics. Research indicates that nanoparticles can achieve enhanced effectiveness when combined with conventional antifungal medications. These nanoparticles can be synthesized in the laboratory using minerals, mineral oxides, and polymers such as silver, selenium, zinc oxide, copper, and magnesium oxide, among others [134]. Additionally, biogenic nanoparticles derived from living organisms, including plants, are also utilized [135]. Antifungal nanoparticles are applied independently or in conjunction with carrier polymers, often in the form of nanocomposites, to amplify their therapeutic impact [136]. According to Safaei et al. (2019), alginate–copper oxide nanoparticles exhibit superior antifungal properties compared to copper oxide nanoparticles used alone [103].

**Figure 7:**
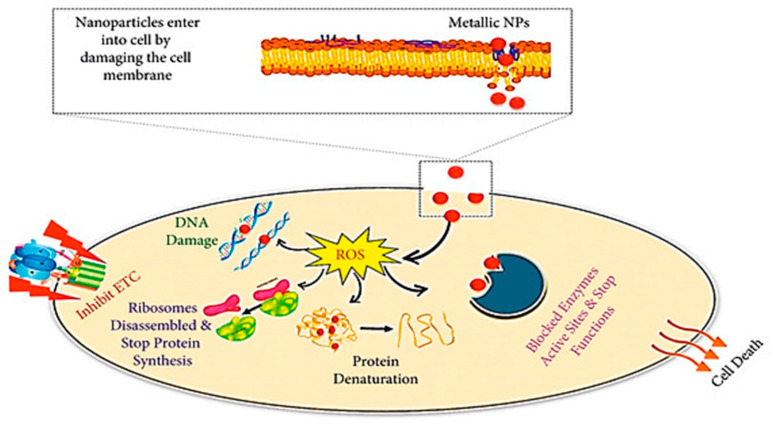
Mechanism of antifungal activity of nanoparticles. Reproduced with permission from [137], copyright 2023 Wiley (CC BY 4.0 DEED).

The physical properties of nanoparticles utilized in nanoparticle composites significantly influence their antimicrobial efficacy [103]. Typically, antimicrobial nanoparticles are employed at sizes below 10 nm, with smaller nanoparticles demonstrating enhanced and quicker effects against pathogenic microorganisms [99]. Moreover, the concentration of the stabilizing carrier polymer plays a crucial role in determining the antimicrobial activity of the prepared nanocomposite, as highlighted by the same researchers. Another study emphasized that the concentration of nanoparticles used also impacts the antifungal effectiveness of nanoparticle composites [104]. These nanomaterials exert their antifungal effects through various mechanisms, including the generation of reactive oxygen species, release of ions, disruption of cell membranes, inhibition of protein synthesis and DNA damage, interference with mitochondrial function, destruction of fungal hyphae, suppression of sporulation, and the prevention of biofilm formation, all of which contribute to their fungicidal properties (Figure 7) [134,137].

Silver nanoparticles are well known for their relatively safe profile on mammalian cells, causing minor cytotoxic and genotoxic effects [97], while exhibiting broad-spectrum antimicrobial properties, including antifungal effects [104]. These nanoparticles can disrupt fungal cell membranes, induce the generation of reactive oxygen species, and interfere with cellular processes. AgNPs have demonstrated efficacy against various fungal pathogens, such as *Candida* and *Aspergillus* species [138,139]. Furthermore, their small size facilitates enhanced penetration and distribution, thereby improving their therapeutic potential [138,139].

Cellulose nanoparticles, known as “nanocellulose,” represent another class of antifungal biomimetics found in wood and plants, or of bacterial origin [140]. Although they exhibit modest direct antifungal effects, they serve as effective carriers for other antifungal agents, thereby regulating their delivery and enhancing their efficacy. For instance, Terea et al. (2023) developed zinc oxide nanoparticles on cellulose nanocrystals, demonstrating excellent antifungal effects against *C. albicans* [141].

Another example is curcumin nanoparticles, which have demonstrated promising antifungal activities. Curcumin, a natural polyphenol extracted from turmeric, exhibits broad-spectrum antimicrobial properties, including antifungal activity [142]. However, its clinical utility is hampered by its poor solubility and bioavailability. Curcumin-loaded nanoparticles address these challenges by enhancing its stability and enabling targeted delivery [143]. For example, curcumin-coated silver nanoparticles have shown significant antifungal efficacy and hold potential for combating fungal infections, particularly those caused by azole-resistant strains of *Aspergillus* species, *Candida* species, and dermatophytes [144,145]. These nanoparticles can be engineered to release curcumin in a controlled manner, optimizing therapeutic outcomes while minimizing side effects [146].

Additionally, other types of nanoparticles such as nanoantimicrobial peptides [147], nanopropolis [148], and chitosan nanoparticles [149] have also demonstrated potent antifungal activities. These advancements underscore their growing importance in the field of antifungal biomimetics research, promising novel approaches to combat fungal infections in the foreseeable future.

### 4.5. Plant-Derived Polyphenols

**Figure 8:**
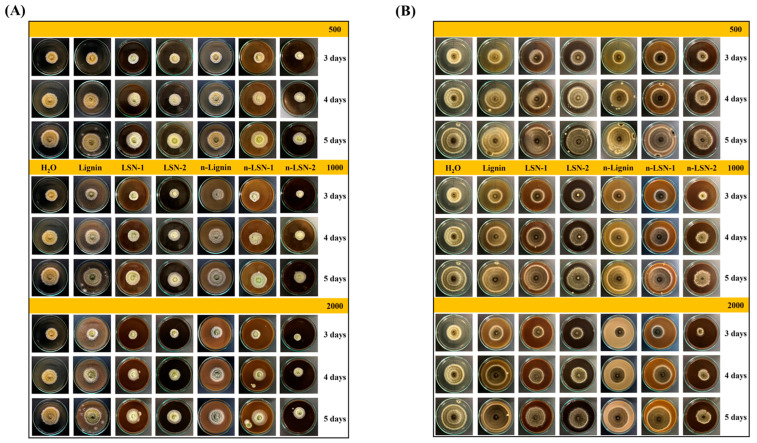
Antifungal effects of rice husk-derived materials against *Aspergillus flavus* (**A**) and *Aspergillus niger* (**B**). The materials tested include lignin extracted from rice husk, nanoparticles made from lignin (n-lignin), and combinations of these nanoparticles capped with silver nanoparticles (LSN-1, LSN-2, n-LSN-1, n-LSN-2). The effectiveness of these materials against the fungi was evaluated over three days (days 3, 4, and 5) at different concentrations (500, 1000, and 2000 μg per 100 μL). Reproduced with permission from [162], copyright 2023 American Chemical Society (CC-BY-NC-ND 4.0).

Plant-derived polyphenols, such as resveratrol and quercetin, exhibit antifungal properties through multiple mechanisms, including the disruption of fungal cell membranes and the inhibition of fungal enzymes [150]. These polyphenols can be formulated into nanoparticles or liposomes to enhance their stability and bioavailability [151]. Clinical studies have demonstrated the antifungal efficacy of polyphenols against a range of pathogens, including *Candida* and *Aspergillus* species [152,153]. Flavonoids, such as quercetin and catechins, are well-documented for their antifungal effects. Quercetin, found in onions, apples, and berries, has shown inhibitory effects against various fungal pathogens, including *C. albicans* and *Aspergillus* species. A study demonstrated that quercetin disrupts fungal cell membrane integrity and inhibits hyphal growth, which is crucial for fungal pathogenicity [154,155,156]. Caffeic acid and ferulic acid are prominent examples of phenolic acids with notable antifungal activity. Caffeic acid, present in coffee, fruits, and vegetables, has been reported to exhibit antifungal effects against *C. albicans* by inducing oxidative stress and damaging fungal cell membranes [157,158]. Similarly, ferulic acid, found in cereals and grains, has shown antifungal activity by damaging the cell integrity and causing the leakage of the cellular content *Fusarium graminearum* [159]. Another example is tannins, such as tannic acid, which are polyphenolic compounds found in tea, nuts, and berries. Tannic acid has demonstrated antifungal activity against *Penicillium digitatum* via disruption of the cell wall and the plasma membrane of the pathogen [160]. Lignans, such as pinoresinol, are another example of the plant-derived polyphenols found in flaxseeds and sesame seeds that also exhibit antifungal properties. For instance, lignans such as pinoresinol and secoisolariciresinol, found in wheat grains, inhibit radial growth and reduce trichothecene levels in five strains of *F. graminearum* [161]. Recent reports also tried to evaluate the use of polyphenol-based nanohybrid materials against important fungal pathogens, especially those that affect agricultural goods. For instance, Vo et al., 2023 evaluated the antifungal activities of rice husk-extracted lignin, nanolignin (n-Lignin), and lignin/n-lignin-capped silver nanoparticles (LSN-1, LSN-2, n-LSN-1, n-LSN-2) on *A. flavus* and *A. niger* [162]. The results confirmed that the hybrid biomaterials (LSN, n-LSN) effectively prevent the growth or generation of fungal spores for both *A. flavus* (Figure 8A) and *A. niger* (Figure 8B).

### 4.6. Probiotic Bacteria

Probiotic bacteria, particularly strains of *Lactobacillus* and *Bifidobacterium*, have demonstrated significant antifungal activity, making them promising candidates for the prevention and treatment of fungal infections. These beneficial bacteria can produce antifungal substances and modulate the host immune response, providing a natural means of combating fungal infections [163]. These bacteria can inhibit the growth of pathogenic fungi by producing organic acids, hydrogen peroxide, and bacteriocins [163]. Clinical studies have shown that probiotic treatments can reduce the incidence and severity of fungal infections, particularly in immunocompromised patients [164].

*Lactobacillus* species are among the most extensively studied probiotics for their antifungal properties. *Lactobacillus rhamnosus* GG, for instance, has shown effectiveness against *Candida* species [165]. Other studies have shown that *L. rhamnosus* and *L. plantarum* strains exhibit an inhibitory effect on the filamentation and biofilm formation of *C. albicans* and *C. tropicalis* [166]. This effect is likely mediated by the metabolites secreted into the culture medium. In another example, *L. plantarum* KCC-10 inhibited the growth of *Epidermophyton floccosum* (KACC 44,918), *Trichophyton roseum* (KACC 40,956), and *T. mentagrophytes* (KACC 45,479), confirming that the antifungal agent produced by this isolate was identified as 3-phenyllactic acid [167].

*Bifidobacterium* has also been shown to exhibit antifungal activity. Studies show that a specific strain of *Bifidobacterium*, *B. adolescentis*, is particularly effective against *C. albicans* [168]. Lab experiments suggest bacteria releases substances that stop *C. albicans* from growing [168]. Additionally, probiotics can even boost the effectiveness of antifungal medications. For instance, combining a probiotic containing *B. longum*, *L. bulgaris*, and *Streptococcus thermophilus* with the antifungal medication nystatin was more effective in the treatment of *Candida*-associated stomatitis than standard therapy alone [169].

### 4.7. Graphene-Based Materials

Graphene-based materials (GBMs), such as graphene oxide (GO) and reduced graphene oxide (rGO), have shown significant promise as antifungal agents due to their unique physicochemical properties [170]. GO, with its abundant oxygen-containing functional groups, and rGO, with its enhanced conductivity and reduced oxygen content, have been extensively studied for their antimicrobial properties, including their effects on fungal pathogens [170,171,172,173,174,175]. Several studies have demonstrated the effectiveness of these materials against a variety of fungal species, highlighting their potential for clinical and industrial applications.

For instance, GO nanosheets have been found to be highly effective against *C. albicans*, a common fungal pathogen responsible for infections such as thrush and systemic candidiasis [176]. Similarly, rGO has been shown to inhibit the growth of *Aspergillus* species, a fungus that can cause severe respiratory infections, especially in immunocompromised individuals [177]. Moreover, graphene-based composites, such as GO functionalized with silver nanoparticles (GO–Ag), have exhibited enhanced antifungal activity [172]. These composites leverage the synergistic effects of GO and silver nanoparticles to effectively inhibit the growth of *C. albicans* and *C. tropicalis*, and other pathogenic fungi [172].

**Figure 9:**
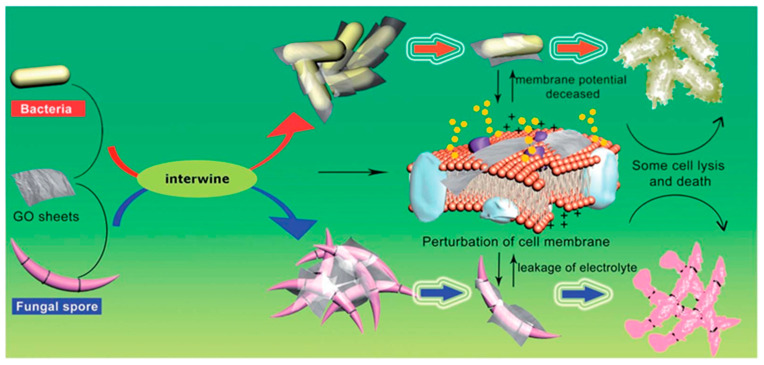
Mechanistic insights into GO-mediated pathogen disruption. This figure depicts the interaction of graphene oxide (GO) sheets with bacterial and fungal cell membranes, resulting in membrane perturbation. Bacteria further experience a loss of membrane potential, while fungal spores exhibit electrolyte leakage. These combined effects ultimately lead to pathogen cell death. Reproduced with permission from [175], copyright 2014 Royal Society of Chemistry.

The antifungal mechanisms of graphene-based materials are multifaceted, involving both physical and chemical interactions with fungal cells. One primary mechanism is the physical disruption of fungal cell membranes. In this mechanism, GO interacts with the pathogens by mechanically wrapping and locally damaging the cell membrane, finally causing cell lysis and death (Figure 9) [175]. This physical interaction is particularly effective against the robust cell walls of fungi, which are typically more resistant to chemical treatments alone.

In addition to physical disruption, graphene-based materials induce oxidative stress within fungal cells. GO and rGO can generate reactive oxygen species (ROS) when in contact with fungal cells. These ROS cause oxidative damage to vital cellular components, including proteins, lipids, and DNA, ultimately leading to cell death [178]. The oxidative stress mechanism is particularly potent because it not only damages the cell membrane but also disrupts intracellular processes, leading to a comprehensive antifungal effect.

The combined effect of GO and various nanoparticles can be utilized to create more effective antimicrobial agents [179,180]. Consequently, there has been a surge of interest in silver (Ag) nanocomposites among researchers in recent years. To enhance the antifungal properties of carbon nanoscrolls (CNSs), Li et al. filled them with silver nanoparticles (AgNPs) and compared their antifungal efficacy to that of GO–AgNPs nanocomposites [172]. The CNSs–AgNPs demonstrated prolonged antifungal activity against *C. albicans* and *C. tropicalis* compared to the GO–AgNP nanocomposites. The results indicated that while GO alone did not produce any inhibition zones, the GO–AgNP samples exhibited clear inhibition zones, demonstrating significant antifungal activity.

Another approach involves combining GBMs with other antifungal agents. For instance, research suggests that graphene oxide–borneol (GOB) composites exhibit strong antifungal activity against *C. albicans*, a common fungal pathogen. The mechanism behind this enhanced effect is thought to involve a combination of factors. The GO component likely disrupts the fungal membrane, while borneol, a naturally occurring antifungal compound, can further damage the fungus and inhibit its growth [181]. These findings suggest that GBMs have the potential to act alone or be synergistically combined with existing antifungal treatments.

Another promising application of graphene-based materials is their use in antifungal coatings and films. GO and rGO can be incorporated into coatings for medical devices and surfaces to prevent fungal colonization and biofilm formation. Studies have demonstrated that GO coatings on catheters can significantly reduce biofilm formation by *C. albicans*, thereby reducing the risk of catheter-related infections [182,183]. These coatings can be particularly beneficial in hospital settings, where fungal infections are a major concern.

### 4.8. Other Biomimetic Antifungal Agents

#### 4.8.1. Essential Oils

**Figure 10:**
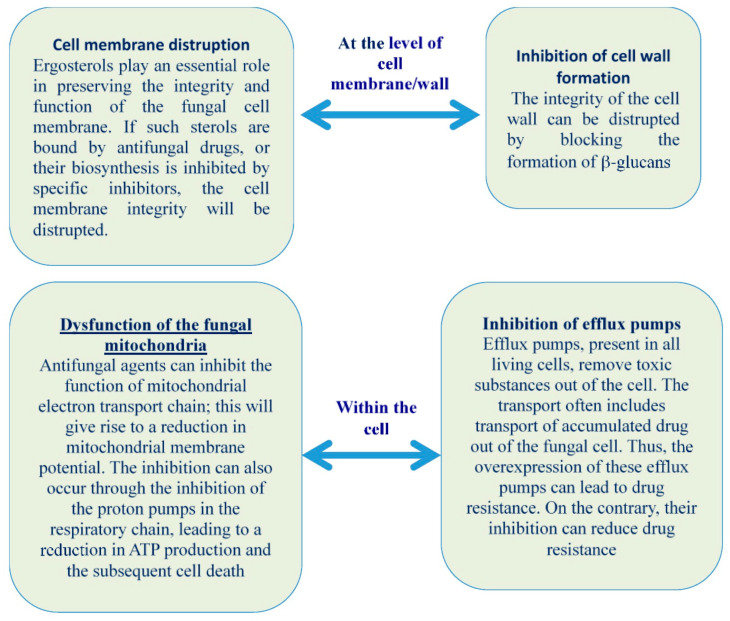
Mechanistic insights into the activity of essential oils against fungal cells. Essential oils inhibit cell wall formation and disrupt cell membranes at cell membrane/wall levels. Within the cells, essential oils inhibit efflux pumps and cause mitochondrial dysfunction in fungi. Reproduced with permission from [184], copyright 2017 MDPI (CC BY 4.0 DEED).

Alongside the previously discussed biomimetic antifungals, other biomimetic groups have also demonstrated promising antifungal activities. A notable example is essential oils, which are extracted from various plants and are rich in compounds with potent antifungal properties [184]. These oils inhibit cell wall formation and disrupt cell membranes. Within the cells, essential oils inhibit efflux pumps and cause mitochondrial dysfunction in fungi (Figure 10) [184]. For instance, tea tree oil, rich in terpinen-4-ol, has shown strong antifungal activity against *Candida* species [185]. Similarly, oregano oil, containing carvacrol and thymol, has been shown to be effective against a range of fungal pathogens [186].

#### 4.8.2. Enzymatic Treatments

Enzymatic treatments, which utilize enzymes such as chitinases and glucanases, represent another example of biomimetic antifungals, offering a targeted approach to antifungal therapy [187,188]. These enzymes degrade the structural components of fungal cell walls, leading to cell lysis. Chitinases, for instance, break down chitin, a major component of fungal cell walls, making them effective against a wide range of fungi [189]. Enzymatic treatments can be used alone or in combination with other antifungal agents to enhance their efficacy [190].

#### 4.8.3. Lysozyme

**Figure 11:**
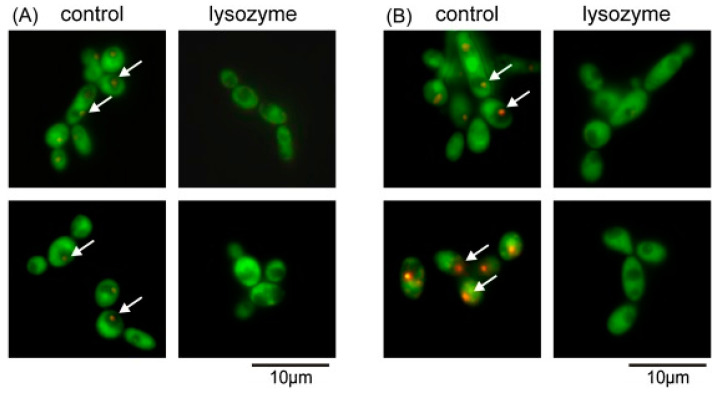
The effect of lysozyme from *G. mellonella* on metabolic activity of *C. albicans*. To assess cell viability, cells were incubated for 1 h (**A**) and 3 h (**B**) at 37 °C in the absence (control) or presence of 0.5 M lysozyme. Following incubation, cells were labeled with a viability staining kit and visualized using high-resolution microscopy. The presence of fluorescent structures within vacuoles (indicated by white arrows) signifies viable cells. Reproduced with permission (License Number 5821880927456) from [193], copyright 2016 Elsevier B.V.

Lysozyme is an enzyme naturally found in human secretions like tears and saliva, as well as other sources, and is known for its ability to break down bacterial cell walls. Its antifungal properties are also well documented [191,192]. Lysozyme works by hydrolyzing the β-1,4-glycosidic bonds in the polysaccharides of fungal cell walls, leading to cell lysis and death [191]. For instance, the N-terminal domain of human milk lysozyme, when treated with pepsin, produced an N-terminal helix that exhibited potent antimicrobial activity. This helix demonstrated significant bactericidal effects against both Gram-positive and Gram-negative bacteria, as well as the fungus *C. albicans*, suggesting its potential for treating infectious diseases [192]. Additionally, the c-type lysozyme from *Galleria melonella* has shown antifungal activity against *C. albicans*, with the mechanism of action having been investigated by a Polish research group in 2016 (Figure 11) [193]. Moreover, a 2017 in vitro study examined the effects of lysozyme on *C. albicans* biofilm formation, revealing that at low concentrations (<30 μg/mL), lysozyme reduced the attached biomass, while at higher concentrations (>300 μg/mL), it promoted biofilm formation [194].

#### 4.8.4. Phospholipid-Based Liposomes

Liposomes are spherical vesicles composed of phospholipid bilayers, widely used as drug delivery systems. They can encapsulate antifungal agents, enhancing their stability and bioavailability while reducing toxicity [195]. The phospholipid bilayers of liposomes can fuse with fungal cell membranes, facilitating the targeted release of the encapsulated drug directly into the fungal cells [196]. Liposomes loaded with amphotericin B, an antifungal drug, have shown enhanced efficacy against *Candida* and *Aspergillus* species while reducing the drug’s nephrotoxicity [197]. These liposomal formulations ensure better penetration of the drug into fungal cells, leading to more effective treatment outcomes.

#### 4.8.5. Propolis

Propolis is a resinous substance produced by honey bees from plant materials. It has a complex chemical composition, including flavonoids, phenolics, and terpenes, which contribute to its antimicrobial properties [198]. Propolis exhibits antifungal activity by disrupting the cell membrane integrity of fungi and inhibiting their enzymatic activity. Studies have shown that propolis extracts effectively inhibit the growth of *Candida* species and *Trichophyton* species, which are common fungal pathogens responsible for local and systemic infections [199]. Propolis can be formulated into creams or ointments for topical application, providing a natural and effective treatment option for fungal infections.

#### 4.8.6. Silk Fibroin-Based Materials

Silk fibroin, a protein derived from the silk of the silkworm *Bombyx mori*, has excellent biocompatibility and mechanical properties [200]. Silk fibroin can be used to create biomaterials with inherent antifungal properties, or as a delivery system for antifungal agents [201,202]. Its structure allows for the sustained release of encapsulated drugs, enhancing their therapeutic effects. For instance, a silk fibroin hydrogel infused with ketoconazole was developed to improve patient compliance [202]. This hydrogel capitalizes on its biocompatibility and biodegradability, and the encapsulated drug exhibited effective antifungal activity against *A. niger* [202]. These films enable a controlled release of the drug, maintaining effective concentrations over extended periods and improving treatment efficacy. 

## 5. Challenges and Future Directions

Biomimetic antifungal materials represent a promising frontier in the battle against multidrug-resistant fungi, a growing concern in medical and agricultural contexts. These materials, inspired by natural antifungal mechanisms found in organisms such as amphibians and plants, offer innovative solutions by mimicking the structural and functional properties that deter fungal growth. However, significant challenges remain, including the complexity of accurately replicating these natural mechanisms at a scalable and cost-effective level [203]. Additionally, ensuring the biocompatibility and environmental safety of these materials is crucial. Here we will explore the major limitations of the most commonly used biomimetic antifungal materials (Table 1).

### 5.1. Antifungal Peptides Limitations

AFPs have garnered significant attention as potential treatments for multidrug-resistant fungi due to their unique mechanisms of action, which differ from conventional antifungals. However, several limitations impede their clinical application (Table 1). One of the primary challenges is their susceptibility to proteolytic degradation, which reduces their stability and efficacy in vivo [203]. Peptides are easily broken down by proteases in the body, leading to a short half-life and necessitating frequent administration or high doses, which can be impractical and costly [204]. Another critical limitation is toxicity. While AFPs are designed to target fungal cells, they can also exhibit cytotoxic effects on mammalian cells. This off-target toxicity can result in undesirable side effects and limit the therapeutic window of these peptides [205]. Additionally, the development of resistance against AFPs, although slower than with traditional antifungals, is still a concern [206]. Fungi can adapt over time, potentially through mutations or alterations in membrane composition, diminishing the peptides’ effectiveness. The delivery of AFPs to the site of infection poses another significant hurdle. Effective targeting and penetration of these peptides into infected tissues remains challenging, especially in systemic infections. This issue is compounded by the peptides’ poor bioavailability and the need for delivery systems that can protect and release them in a controlled manner [207]. Lastly, the cost of production is a substantial barrier. Synthesizing AFPs at a scale necessary for widespread clinical use is expensive, partly due to the complexity of their structures and the precision required in their manufacture [208]. Overcoming these limitations will require advancements in peptide design, delivery technologies, and manufacturing processes, as well as comprehensive clinical trials to ensure their safety and efficacy.

### 5.2. Alginate-Based Hydrogels and Other Biomimetic Hydrogels Limitations

Achieving optimal mechanical strength and stability remains a challenge for alginate-based and other biomimetic hydrogels (Table 1). Alginate hydrogels, while biocompatible and capable of maintaining a moist environment conducive to healing, often suffer from poor mechanical properties, limiting their durability and structural integrity under physiological conditions [209,210]. This issue can lead to premature degradation and reduced effectiveness in sustained drug delivery. Another critical limitation is the controlled release of antifungal agents. While hydrogels are designed to provide a sustained release, achieving the precise control required to maintain therapeutic levels of antifungal agents over extended periods remains difficult [211,212]. Variability in the rate of drug release can lead to suboptimal therapeutic outcomes, either by releasing too little of the drug, which can be ineffective, or too much, which can cause toxicity. The potential for immune response and biocompatibility issues also poses significant challenges. Although alginate and other biomimetic hydrogels are generally considered biocompatible, the risk of immunogenic reactions cannot be entirely eliminated [213]. This risk is particularly relevant for hydrogels incorporating additional bioactive molecules or synthetic components to enhance their properties, which can introduce new variables affecting the body’s immune response. Moreover, the effectiveness of these hydrogels can be compromised by biofilm formation. Multidrug-resistant fungi are adept at forming biofilms, which protect them from antifungal agents [214]. Hydrogels must therefore not only deliver antifungal drugs but also penetrate and disrupt biofilms, a task that remains technically challenging. Finally, scalability and cost are practical barriers to widespread clinical use. The production of alginate-based and other biomimetic hydrogels involves complex processes that can be expensive and difficult to scale up [215]. This economic aspect can limit their accessibility and application in resource-limited settings, where the burden of fungal infections is often highest.

### 5.3. Chitosan and Chitosan Derivatives Limitations

A critical factor influencing chitosan’s antifungal efficacy is its inherent variability, as demonstrated in Table 1. This variability is driven by several factors, including molecular weight, degree of deacetylation, and environmental pH. This variability makes it difficult to standardize chitosan-based treatments and predict their effectiveness consistently [121]. Furthermore, while chitosan has demonstrated in vitro efficacy against a range of fungi, translating these results to in vivo systems has proven problematic, often due to the complex interactions within biological environments [216]. Another significant limitation is solubility. Chitosan is only soluble in acidic conditions (pH < 6.5) [217], which restricts its use in the neutral or alkaline environments typically found in the human body. This solubility issue also complicates the formulation and delivery of chitosan-based antifungals. To address this, researchers have developed various chitosan derivatives with improved solubility profiles [218], but these modifications can sometimes compromise the material’s antifungal efficacy or introduce new biocompatibility concerns. Additionally, the mechanism of action of chitosan against fungi is not fully understood, which complicates efforts to optimize its use. Proposed mechanisms include the disruption of cell membranes, interference with nutrient uptake, and penetrating the cell walls of fungi and binding to its DNA, but the relative contribution of each mechanism remains unclear [149]. This lack of detailed understanding hinders the rational design of more effective chitosan-based antifungals. Biocompatibility and potential cytotoxicity also pose challenges. While chitosan is generally regarded as safe, high concentrations or certain derivatives can exhibit cytotoxic effects on mammalian cells [219]. Ensuring that chitosan-based treatments are both effective against fungi and safe for human cells is crucial, and requires careful balance and rigorous testing. Finally, the production and scalability of chitosan-based antifungal agents are hindered by economic and practical issues. High-quality chitosan must be sourced from chitin, which is primarily derived from shellfish. This raises concerns about sustainability, allergenicity, and batch-to-batch consistency [113]. Scaling up production to meet clinical demand without compromising quality or affordability remains a significant hurdle.

### 5.4. Nanoparticles Limitations

A significant concern associated with nanoparticles is their potential toxicity (Table 1). For instance, AgNPs, despite their broad-spectrum antifungal activity, can cause cytotoxicity and oxidative stress in human cells at higher concentrations [220]. Similarly, zinc oxide nanoparticles (ZnONPs) have demonstrated antifungal activity, but their use is limited due to concerns about their potential to induce cytotoxic effects and DNA damage [221,222]. Ensuring the biocompatibility of nanoparticles while maintaining their antifungal efficacy is crucial for their safe application. The stability of nanoparticles in biological environments is another significant issue. Nanoparticles tend to aggregate in physiological conditions, which can reduce their efficacy and alter their distribution. For example, gold nanoparticles (AuNPs) are prone to aggregation, which can compromise their antifungal activity and lead to inconsistent therapeutic outcomes [223]. Developing strategies to stabilize nanoparticles in biological fluids without affecting their activity remains a challenge. The production of nanoparticles on a large scale with consistent quality and functional properties poses practical challenges. For instance, producing quantum dots (QDs) with uniform size and surface characteristics is technically demanding and costly, limiting their practical application in antifungal therapy [224]. Scaling up the production process without compromising the quality and efficacy of the nanoparticles is essential for their clinical use. The regulatory landscape for nanoparticles is still evolving, with concerns about their long-term environmental impact and human health effects. The use of metal-based nanoparticles, for example, raises questions about their persistence in the environment and potential to cause ecological harm [225]. Comprehensive risk assessments and regulatory frameworks are needed to address these concerns and ensure their safe use.

### 5.5. Plant-Derived Polyphenols Limitations

One of the primary challenges with polyphenols is their poor bioavailability (Table 1). Many polyphenols, such as curcumin and quercetin, have limited solubility in water and are rapidly metabolized in the body, which reduces their therapeutic effectiveness [226]. For example, curcumin, derived from turmeric, exhibits strong antifungal activity in vitro but suffers from rapid degradation and poor absorption in vivo [227]. Enhancing the bioavailability and stability of polyphenols through formulation strategies such as encapsulation in nanoparticles or liposomes is an area of active research, but this remains a challenge. Furthermore, the antifungal efficacy of polyphenols can vary widely depending on the fungal species and strains. For instance, resveratrol, a polyphenol found in grapes, has demonstrated antifungal activity against *C. albicans* but its effectiveness can be inconsistent with other *Candida* species [228]. This variability makes it difficult to predict and standardize treatment outcomes. While polyphenols are generally considered safe, high doses or prolonged use can lead to toxicity and side effects. For example, high concentrations of epigallocatechin gallate (EGCG), a polyphenol in green tea, have been associated with liver toxicity, nephrotoxicity, and gastrointestinal disorders in some cases [229]. The exact mechanisms by which polyphenols exert their antifungal effects are not fully understood. Polyphenols are believed to disrupt fungal cell membranes, inhibit enzymes, and interfere with biofilm formation, but these mechanisms can vary between different polyphenols and fungal species [230]. This lack of detailed understanding complicates efforts to optimize their use and develop more targeted therapies. Although polyphenols are less likely to induce resistance compared to conventional antifungals, the potential for fungi to develop resistance over time, especially with long-term use, is questionable. Continuous monitoring and combination strategies may be required to mitigate this risk. Balancing antifungal efficacy with potential toxicity is crucial for developing safe therapeutic applications. Effective delivery of polyphenols to the site of infection poses another significant challenge. Polyphenols need to be formulated in a way that ensures their stability, bioavailability, and targeted delivery. For example, incorporating polyphenols into hydrogels or polymeric carriers can improve their delivery, but these approaches require further development and validation [231].

### 5.6. Graphene-Based Materials Limitations

The potential toxicity of GBMs emerges as a critical safety consideration (Table 1). Studies have shown that GO and rGO can induce cytotoxicity in mammalian cells, leading to oxidative stress, inflammation, and cell membrane damage [232]. For example, Chang et al. (2011) found that GO caused dose-dependent toxicity in human fibroblasts and lung epithelial cells. Ensuring the biocompatibility of GBMs while maintaining their antifungal efficacy is crucial for safe therapeutic applications [233]. While functionalization of graphene can enhance its solubility and biocompatibility, it can also affect the material’s antifungal properties. Functionalized graphene may exhibit altered interactions with fungal cells, potentially reducing its effectiveness. Additionally, maintaining the stability of functionalized GBMs in physiological conditions is challenging. For instance, functionalized GO tends to aggregate in biological fluids, which can decrease its bioavailability and therapeutic efficiency [234]. The production and disposal of GBMs raise concerns about their environmental impact. Graphene materials can persist in the environment and potentially cause ecological harm and their long-term effects on soil and water ecosystems are not fully understood, posing a barrier to their widespread use [235]. The high cost of production and challenges in scaling up the synthesis of high-quality GBMs limit their practical application. Despite significant advancements in graphene preparation techniques, the development of cost-effective methods for producing large-area monolayers remains immature. The need for high-purity graphene continues to impede large-scale production and commercial applications [236].

### 5.7. Probiotics Limitations

The antifungal activity of probiotics is highly strain-specific, meaning that not all strains of a particular species will exhibit the same effectiveness against fungi. For instance, Jørgensen et al. 2022, confirmed that *Lactobacillus rhamnosus* had significant strain-dependent variations in their antifungal capacity in a pH-dependent mode [237]. This variability necessitates careful selection and characterization of probiotic strains, complicating their standardization and use in treatment protocols. Ensuring that probiotic bacteria survive gastrointestinal transit and successfully colonize the host’s gut is a significant challenge. Factors such as stomach acidity, bile salts, and the existing microbiota can impact the survival and colonization of probiotics [238]. Determining the optimal dosage and delivery method for probiotics is complex. Probiotics must be administered in sufficient quantities to exert a therapeutic effect, but the required dose can vary widely depending on the strain and the fungal infection being targeted [239]. Additionally, different delivery formats, such as capsules and fermented foods, can affect the viability and efficacy of the probiotics. The precise mechanisms by which probiotics exert antifungal effects are not fully understood, which complicates efforts to optimize their use. Probiotics are believed to inhibit fungi through mechanisms such as competition for nutrients and adhesion sites, production of antimicrobial compounds, and modulation of the host immune response [240,241]. However, these mechanisms can vary between different probiotic strains and fungal species, making it difficult to predict their efficacy in different clinical contexts. Although probiotics are generally considered safe, there are potential risks, particularly for immunocompromised individuals. Cases of probiotic-related infections, such as *Lactobacillus* bacteremia, have been reported [242]. Regulatory frameworks for probiotics are still evolving, and ensuring the safety and efficacy of probiotic products requires rigorous testing and quality control. The interaction between administered probiotics and the host’s existing microbiota is complex and not fully understood. Probiotics may not always integrate seamlessly into the host’s microbiota and can sometimes disrupt the balance of the microbial community [243].

## 6. Research Gaps in the Biomimetic Antifungal Materials

Biomimetic antifungal materials represent a promising frontier in combating multidrug-resistant fungi. However, several research gaps need to be addressed to fully harness their potential.

### 6.1. Mechanisms of Action

While biomimetic materials, such as peptides and hydrogels, have shown efficacy against MDR fungi, the precise mechanisms through which they exert their antifungal effects are not fully understood. Comprehensive studies are needed to elucidate these mechanisms at the molecular level, which could lead to the design of more effective materials. Understanding how these materials interact with fungal cell membranes, biofilms, and intracellular targets is crucial [57].

### 6.2. Optimization of Biocompatibility and Toxicity

One major challenge is balancing the antifungal efficacy of biomimetic materials with their biocompatibility and minimizing their toxicity to human cells. For example, while chitosan and its derivatives have shown promising antifungal properties, their biocompatibility varies with the degree of deacetylation and molecular weight [149,218,244]. Further research is required to optimize these parameters to enhance therapeutic indices.

### 6.3. Long-Term Efficacy and Resistance Development

The long-term efficacy of biomimetic antifungal materials and their potential to induce resistance in fungi are areas that need more investigation. There is a need for longitudinal studies to monitor the effectiveness of these materials over extended periods and in diverse clinical settings. Additionally, research should focus on understanding how fungi might adapt to these materials and developing strategies to prevent resistance [245].

### 6.4. Scalability and Cost-Effectiveness

The production of biomimetic antifungal materials at a scale that is sufficient for widespread clinical use remains a significant hurdle. Research into scalable and cost-effective manufacturing processes is essential. This includes exploring alternative sources of raw materials, such as renewable resources, and developing more efficient synthesis methods [246,247].

### 6.5. In Vivo Studies and Clinical Trials

While in vitro studies provide valuable insights, the real-world applicability of biomimetic antifungal materials can only be confirmed through in vivo studies and clinical trials. There is a gap in terms of translating the promising results from laboratory settings to animal models and, subsequently, human trials. More research is needed to evaluate the safety, efficacy, and pharmacokinetics of these materials in living organisms [185,219,223].

### 6.6. Multifunctional and Hybrid Materials

The development of multifunctional and hybrid biomimetic materials that can simultaneously target different aspects of fungal infections—such as biofilm disruption, immune modulation, and direct antifungal activity—represents an emerging area of interest. Research should explore the synergistic effects of combining various biomimetic approaches to enhance overall antifungal efficacy [57,144].

### 6.7. Environmental Impact and Degradation

The environmental impact of biomimetic antifungal materials, particularly their degradation products, is an area that requires further research. Understanding the environmental fate and potential ecological risks associated with the widespread use of these materials is essential for sustainable development [216,225,235].

## 7. Conclusions

In an era marked by the rise of public health threats such as antifungal resistance [1,2,3], antimicrobial resistance [248,249,250,251,252,253], and COVID-19 [254], coupled with a slowdown in the development of new antimicrobials, biomimetics have emerged as promising therapies. Addressing the complexity and adaptability of multi-drug-resistant fungi is crucial, as traditional antifungal drugs are becoming increasingly ineffective. Biomimetic antifungal materials, which mimic natural biological mechanisms, offer targeted and efficient antifungal action. This review discussed various biomimetic agents, including antifungal peptides, chitosan derivatives, nanoparticles, and plant-derived polyphenols, each leveraging unique mechanisms to combat fungi. However, challenges remain in terms of ensuring biocompatibility, optimizing delivery methods, and overcoming potential resistance. Future research should focus on enhancing the stability and efficacy of these materials, integrating multifunctional approaches, and developing sophisticated delivery systems. Interdisciplinary research is essential to understand interactions with fungal cells and the host environment. Addressing research gaps, such as long-term effects on health and the environment, precise mechanisms of action, and standardized testing protocols, is crucial. While biomimetic antifungal materials offer a revolutionary approach to combating resistant fungi, extensive research and development are needed to realize their full potential through collaborative efforts.

## Figures and Tables

**Table 1:** Major challenges for biomimetic antifungal agents.

Biomimetic Name	Major Challenges	References
Antifungal peptides	Susceptibility to proteolytic degradation, cytotoxicity, development of resistance, delivery to the site of infection, and cost of production	[203,204,205,206,207,208]
Alginate-based hydrogels	Poor mechanical strength, controlled drug release issues, potential immune response and biocompatibility issues, biofilm penetration, scalability, and cost	[209,210,211,212,213,214,215]
Chitosan and chitosan derivatives	Variability in antifungal efficacy, solubility limitations, unclear mechanism of action, potential cytotoxicity, and production scalability	[113,121,216,217,218,219]
Nanoparticles	Potential cytotoxicity, stability in biological environments issues, production scalability, and regulatory and environmental concerns	[220,221,222,223,224,225]
Plant-derived polyphenols	Poor bioavailability, variable in its antifungal efficacy, potential toxicity, unclear mechanisms of action, and delivery to the site of infection challenges	[226,227,228,229,230,231]
Graphene-based materials	Potential cytotoxicity, functionalization affecting properties and stability, environmental impact, and high production cost	[232,233,234,235,236]
Probiotics	Strain-specific efficacy, gastrointestinal survival, optimal dosage, unclear mechanisms of action, potential risks for immunocompromised individuals, interaction with host microbiota, and regulatory frameworks	[237,238,239,240,241,242,243]

## Data Availability

Not applicable.

## References

[1] Khalifa H.O., Watanabe A., Kamei K. (2022). Azole and echinocandin resistance mechanisms and genotyping of *Candida tropicalis* in Japan: Cross-boundary dissemination and animal–human transmission of *C. tropicalis* infection. Clin. Microbiol. Infect..

[2] Khalifa H.O., Hubka V., Watanabe A., Nagi M., Miyazaki Y., Yaguchi T., Kamei K. (2022). Prevalence of antifungal resistance, genetic basis of acquired azole and echinocandin resistance, and genotyping of *Candida krusei* recovered from an international collection. Antimicrob. Agents Chemother..

[3] Khalifa H.O., Watanabe A., Kamei K. (2024). Genetic mutations in *FKS1* gene associated with acquired echinocandin resistance in *Candida parapsilosis* complex. Mycopathologia.

[4] Mudenda S. (2024). Global Burden of fungal infections and antifungal resistance from 1961 to 2024: Findings and future implications. Pharmacol. Pharm..

[5] Perlin D.S., Rautemaa-Richardson R., Alastruey-Izquierdo A. (2017). The global problem of antifungal resistance: Prevalence, mechanisms, and management. Lancet Infect. Dis..

[6] Khalifa H.O., Watanabe A., Kamei K. (2023). Antifungal resistance and genotyping of clinical *Candida parapsilosis* complex in Japan. J. Fungi.

[7] Khalifa H.O., Arai T., Majima H., Watanabe A., Kamei K. (2020). Genetic basis of azole and echinocandin resistance in clinical *Candida glabrata* in Japan. Antimicrob. Agents Chemother..

[8] Denning D. (2024). Global incidence and mortality of severe fungal disease. Lancet Infect. Dis..

[9] World Health Organization (2022). WHO Fungal Priority Pathogens List to Guide Research, Development and Public Health Action.

[10] Negm E.M., Mohamed M.S., Rabie R.A., Fouad W.S., Beniamen A., Mosallem A., Tawfik A.E., Salama H.M. (2023). Fungal infection profile in critically ill COVID-19 patients: A prospective study at a large teaching hospital in a middle-income country. BMC Infect. Dis..

[11] Hoenigl M., Seidel D., Sprute R., Cunha C., Oliverio M., Goldman G.H., Ibrahim A.S., Carvalho A. (2022). COVID-19-associated fungal Infections. Nat. Microbiol..

[12] Hossain C.M., Ryan L.K., Gera M., Choudhuri S., Lyle N., Ali K.A., Diamond G. (2022). Antifungals and drug resistance. Encyclopedia.

[13] Khalifa H.O., Majima H., Watanabe A., Kamei K. (2021). In vitro characterization of twenty-one antifungal combinations against echinocandin-resistant and-susceptible *Candida glabrata*. J. Fungi.

[14] Robbins N., Caplan T., Cowen L.E. (2017). Molecular evolution of antifungal drug resistance. Annu. Rev. Microbiol..

[15] Spitzer M., Robbins N., Wright G.D. (2017). Combinatorial strategies for combating invasive fungal infections. Virulence.

[16] Khalifa H.O., Kamimoto M., Shimamoto T., Shimamoto T. (2015). Antimicrobial effects of blueberry, raspberry, and strawberry aqueous extracts and their effects on virulence gene expression in *Vibrio cholerae*. Phytother. Res..

[17] Baltz R.H. (2009). Daptomycin: Mechanisms of Action and Resistance, and Biosynthetic Engineering. Curr. Opin. Chem. Biol..

[18] Clatworthy A.E., Pierson E., Hung D.T. (2007). Targeting virulence: A new paradigm for antimicrobial therapy. Nat. Chem. Biol..

[19] Gauwerky K., Borelli C., Korting H.C. (2009). Targeting virulence: A new paradigm for antifungals. Drug Discov. Today.

[20] Armstrong-James D., Brown G.D., Netea M.G., Zelante T., Gresnigt M.S., van de Veerdonk F.L., Levitz S.M. (2017). Immunotherapeutic approaches to treatment of fungal diseases. Lancet Infect. Dis..

[21] Sandhu Z.A., Raza M.A., Alqurashi A., Sajid S., Ashraf S., Imtiaz K., Aman F., Alessa A.H., Shamsi M.B., Latif M. (2024). Advances in the optimization of Fe nanoparticles: Unlocking antifungal properties for biomedical applications. Pharmaceutics.

[22] Jangjou A., Zareshahrabadi Z., Abbasi M., Talaiekhozani A., Kamyab H., Chelliapan S., Vaez A., Golchin A., Tayebi L., Vafa E. (2022). Time to conquer fungal infectious diseases: Employing nanoparticles as powerful and versatile antifungal nanosystems against a wide variety of fungal species. Sustainability.

[23] Bigham A., Zarepour A., Safarkhani M., Huh Y., Khosravi A., Rabiee N., Iravani S., Zarrabi A. (2024). Inspired by nature: Bioinspired and biomimetic photocatalysts for biomedical applications. Nano Mater. Sci..

[24] Li T., Li L., Du F., Sun L., Shi J., Long M., Chen Z. (2021). Activity and mechanism of action of antifungal peptides from microorganisms: A review. Molecules.

[25] Pappas P.G., Kauffman C.A., Andes D.R., Clancy C.J., Marr K.A., Ostrosky-Zeichner L., Reboli A.C., Schuster M.G., Vazquez J.A., Walsh T.J. (2016). Clinical practice guideline for the management of candidiasis: 2016 Update by the Infectious Diseases Society of America. Clin. Infect. Dis..

[26] Pfaller M.A. (2012). Antifungal drug resistance: Mechanisms, epidemiology, and consequences for treatment. Am. J. Med..

[27] Howard S.J., Arendrup M.C. (2011). Acquired antifungal drug resistance in *Aspergillus fumigatus*: Epidemiology and detection. Med. Mycol..

[28] Hagen F., Hare Jensen R., Meis J.F., Arendrup M.C. (2016). Molecular epidemiology and in vitro antifungal susceptibility testing of 108 clinical *Cryptococcus neoformans* Sensu Lato and *Cryptococcus gattii* Sensu Lato isolates from Denmark. Mycoses.

[29] Pfaller M.A., Diekema D.J., Gibbs D.L., Newell V.A., Ellis D., Tullio V., Rodloff A., Fu W., Ling T.A. (2010). Results from the ARTEMIS DISK global antifungal surveillance Study, 1997 to 2007: A 10.5-year analysis of susceptibilities of *Candida* Species to fluconazole and voriconazole as determined by CLSI atandardized disk diffusion. J. Clin. Microbiol..

[30] Fisher M.C., Alastruey-Izquierdo A., Berman J., Bicanic T., Bignell E.M., Bowyer P., Bromley M., Brüggemann R., Garber G., Cornely O.A. (2022). Tackling the emerging threat of antifungal resistance to human health. Nat. Rev. Microbiol..

[31] Cowen L.E., Sanglard D., Howard S.J., Rogers P.D., Perlin D.S. (2015). Mechanisms of antifungal drug resistance. Cold Spring Harb. Perspect. Med..

[32] Onishi J., Meinz M., Thompson J., Curotto J., Dreikorn S., Rosenbach M., Douglas C., Abruzzo G., Flattery A., Kong L. (2000). Discovery of novel antifungal (1,3)-β-D-glucan synthase inhibitors. Antimicrob. Agents Chemother..

[33] Cleveland A.A., Farley M.M., Harrison L.H., Stein B., Hollick R., Lockhart S.R., Magill S.S., Derado G., Park B.J., Chiller T.M. (2012). Changes in incidence and antifungal drug resistance in candidemia: Results from population-based laboratory surveillance in Atlanta and Baltimore, 2008–2011. Clin. Infect. Dis..

[34] Lortholary O., Desnos-Ollivier M., Sitbon K., Fontanet A., Bretagne S., Dromer F. (2011). Recent exposure to caspofungin or fluconazole influences the epidemiology of candidemia: A prospective multicenter study involving 2441 patients. Antimicrob. Agents Chemother..

[35] Khalifa H.O., Arai T., Majima H., Watanabe A., Kamei K. (2021). Evaluation of Surveyor Nuclease for rapid identification of *FKS* genes mutations in *Candida glabrata*. J. Infect. Chemother..

[36] Anderson T.M., Clay M.C., Cioffi A.G., Diaz K.A., Hisao G.S., Tuttle M.D., Nieuwkoop A.J., Comellas G., Maryum N., Wang S. (2014). Amphotericin forms an extramembranous and fungicidal sterol sponge. Nat. Chem. Biol..

[37] Delma F.Z., Al-Hatmi A.M., Brüggemann R.J., Melchers W.J., de Hoog S., Verweij P.E., Buil J.B. (2021). Molecular mechanisms of 5-fluorocytosine resistance in yeasts and filamentous fungi. J. Fungi.

[38] Vermes A., Guchelaar H.J., Dankert J. (2000). Flucytosine: A review of its pharmacology, clinical indications, pharmacokinetics, toxicity and drug interactions. J. Antimicrob. Chemother..

[39] Chandra J., Ghannoum M.A., Mayers D.L., Sobel J.D., Ouellette M., Kaye K.S., Marchaim D. (2017). Flucytosine treatment and resistance mechanisms. Antimicrobial Drug Resistance.

[40] Pfaller M., Neofytos D., Diekema D., Azie N., Meier-Krisesche H.-U., Quan S.-P., Horn D. (2012). Epidemiology and outcomes of candidemia in 3648 patients: Data for the prospective antifungal therapy (PATH Alliancew) registry, 2004–2008. Diagn. Microbiol. Infect. Dis..

[41] Herbrecht R., Denning D.W., Patterson T.F., Bennett J.E., Greene R.E., Oestmann J.W., Kern W.V., Marr K.A., Ribaud P., Lortholary O. (2002). Voriconazole versus amphotericin B for primary therapy of invasive aspergillosis. N. Engl. J. Med..

[42] Roemer T., Krysan D.J. (2014). Antifungal drug development: Challenges, unmet clinical needs, and new approaches. Cold Spring Harb. Perspect. Med..

[43] Rex J.H., Walsh T.J., Nettleman M., Anaissie E.J., Bennett J.E., Bow E.J., Carillo-Munoz A.J., Chavanet P., Cloud G.A., Denning D.W. (2001). Need for alternative trial designs and evaluation strategies for therapeutic studies of invasive mycoses. Clin. Infect. Dis..

[44] Boucher H.W., Talbot G.H., Bradley J.S., Edwards J.E., Gilbert D., Rice L.B., Scheld M., Spellberg B., Bartlett J. (2009). Bad bugs, no drugs: No ESKAPE! An update from the Infectious Diseases Society of America. Clin. Infect. Dis..

[45] Rahamim V., Azagury A. (2021). Bioengineered biomimetic and bioinspired noninvasive drug delivery systems. Adv. Funct. Mater..

[46] Bar-Cohen Y. (2006). Biomimetics: Biologically Inspired Technologies.

[47] Das D., Noh I., Noh I. (2018). Overviews of biomimetic medical materials. Biomimetic Medical Materials: From Nanotechnology to 3D Bioprinting.

[48] Del Bakhshayesh A.R., Asadi N., Alihemmati A., Tayefi Nasrabadi H., Montaseri A., Davaran S., Saghati S., Akbarzadeh A., Abedelahi A. (2019). An overview of advanced biocompatible and biomimetic materials for creation of replacement structures in the musculoskeletal systems: Focusing on cartilage tissue engineering. J. Biol. Eng..

[49] Chee E., Brown A.C. (2020). Biomimetic antimicrobial material strategies for combating antibiotic resistant bacteria. Biomater. Sci..

[50] Khalifa H.O., Okanda T., Abd El-Hafeez A.A., Abd El Latif A., Habib A.G., Yano H., Kato Y., Matsumoto T. (2020). Comparative evaluation of five assays for detection of carbapenemases with a proposed scheme for their precise application. J. Mol. Diagn..

[51] Khalifa H.O., Soliman A.M., Saito T., Kayama S., Yu L., Hisatsune J., Sugai M., Nariya H., Ahmed A.M., Shimamoto T. (2020). First report of foodborne *Klebsiella pneumoniae* coharboring *bla*_VIM-1_, *bla*_NDM-1_, and *mcr-9*. Antimicrob. Agents Chemother..

[52] Rajendran N.K., Kumar S.S.D., Houreld N.N., Abrahamse H. (2018). A review on nanoparticle based treatment for wound healing. J. Drug Deliv. Sci. Technol..

[53] Wang J., Vermerris W. (2016). Antimicrobial nanomaterials derived from natural products—A review. Materials.

[54] Fernandez-Moure J.S., Van Eps J.L., Cabrera F.J., Barbosa Z., Del Rosal G.M., Weiner B.K., Ellsworth W.A., Tasciotti E. (2017). Platelet-rich plasma: A biomimetic approach to enhancement of surgical wound healing. J. Surg. Res..

[55] Espina L., Pagán R., López D., García-Gonzalo D. (2015). Individual constituents from essential oils inhibit biofilm mass production by multi-drug resistant *Staphylococcus aureus*. Molecules.

[56] Loc-Carrillo C., Abedon S.T. (2011). Pros and cons of phage therapy. Bacteriophage.

[57] Efremenko E., Aslanli A., Stepanov N., Senko O., Maslova O. (2023). Various biomimetics, including peptides as antifungals. Biomimetics.

[58] Bentz M.L., Nunnally N., Lockhart S.R., Sexton D.J., Berkow E.L. (2021). Antifungal activity of nikkomycin Z against *Candida auris*. J. Antimicrob. Chemother..

[59] Kočendová J., Vaňková E., Volejníková A., Nešuta O., Buděšínský M., Socha O., Hájek M., Hadravová R., Čerovský V. (2019). Antifungal activity of analogues of antimicrobial peptides isolated from bee venoms against vulvovaginal *Candida* spp.. FEMS Yeast Res..

[60] Heymich M.-L., Nißl L., Hahn D., Noll M., Pischetsrieder M. (2021). Antioxidative, antifungal and additive activity of the antimicrobial peptides Leg1 and Leg2 from chickpea. Foods.

[61] Brady D., Grapputo A., Romoli O., Sandrelli F. (2019). Insect cecropins, antimicrobial peptides with potential therapeutic applications. Int. J. Mol. Sci..

[62] Ki M.R., Kim S.H., Park T.I., Pack S.P. (2023). Self-entrapment of antimicrobial peptides in silica particles for stable and effective antimicrobial peptide delivery system. Int. J. Mol. Sci..

[63] Peng C., Liu Y., Shui L., Zhao Z., Mao X., Liu Z. (2022). Mechanisms of action of the antimicrobial peptide cecropin in the killing of *Candida albicans*. Life.

[64] Vizioli J., Bulet P., Charlet M., Lowenberger C., Blass C., Müller H.M., Richman A. (2000). Cloning and analysis of a cecropin gene from the malaria vector mosquito, *Anopheles gambiae*. Insect Mol. Biol..

[65] Lowenberger C., Charlet M., Vizioli J., Kamal S., Richman A., Christensen B.M., Bulet P. (1999). Antimicrobial activity spectrum, cDNA cloning, and mRNA expression of a newly isolated member of the cecropin family from the mosquito vector *Aedes aegypti*. J. Biol. Chem..

[66] Xu X., Yang H., Ma D., Wu J., Wang Y., Song Y., Lai R. (2008). Toward an understanding of the molecular mechanism for successful blood feeding by coupling proteomics analysis with pharmacological testing of horsefly salivary glands. Mol. Cell. Proteom..

[67] Ekengren S., Hultmark D. (1999). Drosophila cecropin as an antifungal agent. Insect Biochem. Mol. Biol..

[68] Boulanger N., Munks R.J., Hamilton J.V., Vovelle F., Brun R., Lehane M.J., Bulet P. (2002). Epithelial innate immunity: A novel antimicrobial peptide with antiparasitic activity in the blood-sucking insect *Stomoxys calcitrans*. J. Biol. Chem..

[69] De Lucca A.J., Bland J.M., Jacks T.J., Grimm C., Walsh T.J. (1998). Fungicidal and binding properties of the natural peptides cecropin B and dermaseptin. Med. Mycol..

[70] Lu D., Geng T., Hou C., Huang Y., Qin G., Guo X. (2016). Bombyx mori cecropin A has a high antifungal activity to entomopathogenic fungus *Beauveria bassiana*. Gene.

[71] Lee E., Kim J.K., Jeon D., Jeong K.W., Shin A., Kim Y. (2015). Functional roles of aromatic residues and helices of papiliocin in its antimicrobial and anti-inflammatory activities. Sci. Rep..

[72] Yoe S.M., Kang C.S., Han S.S., Bang I.S. (2006). Characterization and cDNA cloning of hinnavin II, a cecropin family antibacterial peptide from the cabbage butterfly, *Artogeia rapae*. Comp. Biochem. Physiol. B Biochem. Mol. Biol..

[73] De Lucca A.J., Bland J.M., Vigo C.B., Jacks T.J., Peter J., Walsh T.J. (2000). D-Cecropin B: Proteolytic resistance, lethality for pathogenic fungi and binding properties. Med. Mycol..

[74] Ji S., Li W., Zhang L., Zhang Y., Cao B. (2014). Cecropin A–melittin mutant with improved proteolytic stability and enhanced antimicrobial activity against bacteria and fungi associated with gastroenteritis in vitro. Biochem. Biophys. Res. Commun..

[75] Lee J.K., Seo C.H., Luchian T., Park Y. (2016). Antimicrobial peptide CMA3 derived from the CA-MA hybrid peptide: Antibacterial and anti-inflammatory activities with low cytotoxicity and mechanism of action in *Escherichia coli*. Antimicrob. Agents Chemother..

[76] Silva P.M., Gonçalves S., Santos N.C. (2014). Defensins: Antifungal lessons from eukaryotes. Front. Microbiol..

[77] Wang Z.Z., Shi M., Ye X.Q., Chen M.Y., Chen X.X. (2013). Identification, characterization and expression of a defensin-like antifungal peptide from the Whitefly *Bemisia tabaci* (Gennadius) (Hemiptera: Aleyrodidae). Insect Mol. Biol..

[78] Gao B., del Carmen Rodriguez M., Lanz-Mendoza H., Zhu S. (2017). AdDLP, a Bacterial defensin-like peptide, exhibits anti-plasmodium activity. Biochem. Biophys. Res. Commun..

[79] Gao B., Zhu S. (2012). Alteration of the mode of antibacterial action of a defensin by the amino-terminal loop substitution. Biochem. Biophys. Res. Commun..

[80] de Oliveira Carvalho A., Moreira Gomes V. (2011). Plant defensins and defensin-like peptides-biological activities and biotechnological applications. Curr. Pharm. Des..

[81] Lacerda A.F., Vasconcelos É.A., Pelegrini P.B., Grossi de Sa M.F. (2014). Antifungal defensins and their role in plant defense. Front. Microbiol..

[82] Maisetta G., Di Luca M., Esin S., Florio W., Brancatisano F.L., Bottai D., Campa M., Batoni G. (2008). Evaluation of the inhibitory effects of human serum components on bactericidal activity of human beta defensin 3. Peptides.

[83] McPhee J.B., Scott M.G., Hancock R.E. (2005). Design of host defence peptides for antimicrobial and immunity enhancing activities. Comb. Chem. High Throughput Screen..

[84] Zhao C., Nguyen T., Boo L.M., Hong T., Espiritu C., Orlov D., Wang W., Waring A., Lehrer R.I. (2001). RL-37, an Alpha-helical antimicrobial peptide of the rhesus monkey. Antimicrob. Agents Chemother..

[85] Termen S., Tollin M., Olsson B., Svenberg T., Agerberth B., Gudmundsson G.H. (2003). Phylogeny, processing and expression of the rat cathelicidin rCRAMP: A model for innate antimicrobial peptides. Cell. Mol. Life Sci..

[86] Tomasinsig L., Zanetti M. (2005). The Cathelicidins-structure, function and evolution. Curr. Protein Pept. Sci..

[87] Holani R., Rathnayaka C., Blyth G.A., Babbar A., Lahiri P., Young D., Dufour A., Hollenberg M.D., McKay D.M., Cobo E.R. (2023). Cathelicidins induce toll-interacting protein synthesis to prevent apoptosis in colonic epithelium. J. Innate Immun..

[88] Wang Y., Hong J., Liu X., Yang H., Liu R., Wu J., Wang A., Lin D., Lai R. (2008). Snake cathelicidin from *Bungarus fasciatus* is a potent peptide antibiotic. PLoS ONE.

[89] Broekman D.C., Guðmundsson G.H., Maier V.H. (2013). Differential regulation of cathelicidin in salmon and cod. Fish Shellfish Immunol..

[90] van Dijk A., Molhoek E.M., Bikker F.J., Yu P.-L., Veldhuizen E.J.A., Haagsman H.P. (2011). Avian cathelicidins: Paradigms for the development of anti-infectives. Vet. Microbiol..

[91] Memariani M., Memariani H. (2024). Antifungal properties of cathelicidin LL-37: Current knowledge and future research directions. World J. Microbiol. Biotechnol..

[92] Van Eijk M., Boerefijn S., Cen L., Rosa M., Morren M.J., Van Der Ent C.K., Kraak B., Dijksterhuis J., Valdes I.D., Haagsman H.P. (2020). Cathelicidin-inspired antimicrobial peptides as novel antifungal compounds. Med. Mycol..

[93] Bartels E.J.H., Dekker D., Amiche M. (2019). Dermaseptins, multifunctional antimicrobial peptides: A review of their pharmacology, effectivity, mechanism of action, and possible future directions. Front. Pharmacol..

[94] Nicolas P., El Amri C. (2009). The dermaseptin superfamily: A gene-based combinatorial library of antimicrobial peptides. Biochim. Biophys. Acta Biomembr..

[95] Morton C.O., Dos Santos S.C., Coote P.J. (2007). An Amphibian-derived, cationic, alpha-helical antimicrobial peptide kills yeast by caspase-independent but aif-dependent programmed cell death. Mol. Microbiol..

[96] Belmadani A., Semlali A., Rouabhia M. (2018). Dermaseptin-S1 decreases *Candida albicans* growth, biofilm formation and the expression of hyphal wall protein 1 and aspartic protease genes. J. Appl. Microbiol..

[97] Narayanan K.B., Han S.S. (2017). Dual-crosslinked poly (vinyl alcohol)/sodium alginate/silver nanocomposite beads–a promising antimicrobial material. Food Chem..

[98] Spadari C.D.C., Lopes L.B., Ishida K. (2017). Potential use of alginate-based carriers as antifungal delivery system. Front. Microbiol..

[99] Naseem K., Tahir M.H., Farooqi F., Manzoor S., Khan S.U. (2023). Strategies adopted for the preparation of sodium alginate-based nanocomposites and their role as catalytic, antibacterial, and antifungal agents. Rev. Chem. Eng..

[100] Tomić S.L., Babić Radić M.M., Vuković J.S., Filipović V.V., Nikodinović-Runić J., Vukomanović M. (2023). Alginate-based hydrogels and scaffolds for biomedical applications. Mar. Drugs.

[101] Ahmad N., Bukhari S.N.A., Hussain M.A., Ejaz H., Munir M.U., Amjad M.W. (2024). Nanoparticles incorporated hydrogels for delivery of antimicrobial agents: Developments and trends. RSC Adv..

[102] Gaharwar A.K., Peppas N.A., Khademhosseini A. (2014). Nanocomposite hydrogels for biomedical applications. Biotechnol. Bioeng..

[103] Safaei M., Taran M., Imani M.M. (2019). Preparation, structural characterization, thermal properties and antifungal activity of alginate-CuO bionanocomposite. Mater. Sci. Eng. C.

[104] Xiang S., Ma X., Shi H., Ma T., Tian C., Chen Y., Chen H., Chen X., Luo K., Cai L. (2019). Green synthesis of an alginate-coated silver nanoparticle shows high antifungal activity by enhancing its cell membrane penetrating ability. ACS Appl. Bio Mater..

[105] Gong Y., Han G., Zhang Y., Pan Y., Li X., Xia Y., Wu Y. (2012). Antifungal activity and cytotoxicity of zinc, calcium, or copper alginate fibers. Biol. Trace Elem. Res..

[106] Younes I., Rinaudo M. (2015). Chitin and chitosan preparation from marine sources. Structure, properties and applications. Mar. Drugs.

[107] Streit F., Koch F., Laranjeira M., Ninow J.L. (2009). Production of fungal chitosan in liquid cultivation using apple pomace as substrate. Braz. J. Microbiol..

[108] Song C., Yu H., Zhang M., Yang Y., Zhang G. (2013). Physicochemical properties and antioxidant activity of chitosan from the blowfly *Chrysomya megacephala* larvae. Int. J. Biol. Macromol..

[109] Hamdine M., Heuzey M.C., Bégin A. (2005). Effect of organic and inorganic acids on concentrated chitosan solutions and gels. Int. J. Biol. Macromol..

[110] Hernández-Lauzardo A.N., Bautista-Baños S., Velazquez-Del Valle M.G., Méndez-Montealvo M.G., Sánchez-Rivera M.M., Bello-Perez L.A. (2008). Antifungal effects of chitosan with different molecular weights on in vitro development of *Rhizopus stolonifer* (Ehrenb.: Fr.) Vuill. Carbohydr. Polym..

[111] Li K., Xing R., Liu S., Qin Y., Meng X., Li P. (2012). Microwave-assisted degradation of chitosan for a possible use in inhibiting crop pathogenic fungi. Int. J. Biol. Macromol..

[112] Rahman M.H., Hjeljord L.G., Aam B.B., Sørlie M., Tronsmo A. (2015). Antifungal effect of chito-oligosaccharides with different degrees of polymerization. Eur. J. Plant Pathol..

[113] Verlee A., Mincke S., Stevens C.V. (2017). Recent developments in antibacterial and antifungal chitosan and its derivatives. Carbohydr. Polym..

[114] Chang S.H., Lin H.T.V., Wu G.J., Tsai G.J. (2015). pH Effects on solubility, zeta potential, and correlation between antibacterial activity and molecular weight of chitosan. Carbohydr. Polym..

[115] Shih P.Y., Liao Y.T., Tseng Y.K., Deng F.S., Lin C.H. (2019). A potential antifungal effect of chitosan against *Candida albicans* is mediated via the inhibition of SAGA complex component expression and the subsequent alteration of cell surface integrity. Front. Microbiol..

[116] Galvan Marquez I., Akuaku J., Cruz I., Cheetham J., Golshani A., Smith M.L. (2013). Disruption of protein synthesis as antifungal mode of action by chitosan. Int. J. Food Microbiol..

[117] Palma-Guerrero J., Lopez-Jimenez J.A., Pérez-Berná A.J., Huang I.C., Jansson H.B., Salinas J., Villalaín J., Read N.D., Lopez-Llorca L.V. (2010). Membrane fluidity determines sensitivity of filamentous fungi to chitosan. Mol. Microbiol..

[118] Chung Y.C., Chen C.Y. (2008). Antibacterial characteristics and activity of acid-soluble chitosan. Bioresour. Technol..

[119] Cuero R.G., Duffus E., Osuji G., Pettit R. (1991). Aflatoxin control in preharvest maize: Effects of chitosan and two microbial agents. J. Agric. Sci..

[120] Laflamme P., Benhamou N., Bussières G., Dessureault M. (2000). Differential effect of chitosan on root rot fungal pathogens in forest nurseries. Can. J. Bot..

[121] Rabea E.I., Badawy M.E.T., Stevens C.V., Smagghe G., Steurbaut W. (2003). Chitosan as antimicrobial agent: Applications and mode of action. Biomacromolecules.

[122] Alburquenque C., Bucarey S.A., Neira-Carrillo A., Urzúa B., Hermosilla G., Tapia C.V. (2010). Antifungal activity of low molecular weight chitosan against clinical isolates of *Candida* spp.. Med. Mycol..

[123] Wang Y., Li B., Zhang X., Peng N., Mei Y., Liang Y. (2017). Low molecular weight chitosan is an effective antifungal agent against *Botryosphaeria* sp. and preservative agent for pear (*Pyrus*) fruits. Int. J. Biol. Macromol..

[124] Monga S., Hoang T.X., Park J.K., Kim J.Y. (2020). Antifungal activity of chitosan against *Trichophyton rubrum*. J. Chitin Chitosan.

[125] Ke Y., Ding B., Zhang M., Dong T., Fu Y., Lv Q., Ding W., Wang X. (2022). Study on inhibitory activity and mechanism of chitosan oligosaccharides on *Aspergillus flavus* and *Aspergillus fumigatus*. Carbohydr. Polym..

[126] Peng Y., Han B., Liu W., Xu X. (2005). Preparation and antimicrobial activity of hydroxypropyl chitosan. Carbohydr. Res..

[127] de Oliveira Pedro R., Takaki M., Gorayeb T.C.C., Del Bianchi V.L., Thomeo J.C., Tiera M.J., de Oliveira Tiera V.A. (2013). Synthesis: Characterization and antifungal activity of quaternary derivatives of chitosan on *Aspergillus flavus*. Microbiol. Res..

[128] Tajdini F., Amini M.A., Nafissi-Varcheh N., Faramarzi M.A. (2010). Production, physiochemical and antimicrobial properties of fungal chitosan from *Rhizomucor miehei* and *Mucor racemosus*. Int. J. Biol. Macromol..

[129] Liu X.F., Guan Y.L., Yang D.Z., Li Z., Yao K.D. (2001). Antibacterial action of chitosan and carboxymethylated chitosan. J. Appl. Polym. Sci..

[130] Balicka-Ramisz A., Wojtasz-Pajak A., Pilarckyk B., Ramisz A., Laurans L. Antibacterial and antifugal activity of chitosan. Proceedings of the 12th ISAH Congress on Animal Hygiene.

[131] Zakrzewska A., Boorsma A., Delneri D., Brul S., Oliver S.G., Klis F.M. (2007). Cellular processes and pathways that protect *Saccharomyces cerevisiae* cells against the plasma membrane-perturbing compound chitosan. Eukaryot. Cell.

[132] Saharan V., Mehrotra A., Khatik R., Rawal P., Sharma S.S., Pal A. (2013). Synthesis of chitosan-based nanoparticles and their in vitro evaluation against phytopathogenic fungi. Int. J. Biol. Macromol..

[133] Ziani K., Fernández-Pan I., Royo M., Maté J.I. (2009). Antifungal activity of films and solutions based on chitosan against typical seed fungi. Food Hydrocoll..

[134] Slavin Y.N., Bach H. (2022). Mechanisms of antifungal properties of metal nanoparticles. Nanomaterials.

[135] Hiba H., Thoppil J.E. (2022). Medicinal herbs as a panacea for biogenic silver nanoparticles. Bull. Natl. Res. Cent..

[136] León-Buitimea A., Garza-Cervantes J.A., Gallegos-Alvarado D.Y., Osorio-Concepción M., Morones-Ramírez J.R. (2021). Nanomaterial-based antifungal therapies to combat fungal diseases aspergillosis, coccidioidomycosis, mucormycosis, and candidiasis. Pathogens.

[137] Parveen J., Sultana T., Kazmi A., Malik K., Ullah A., Ali A., Qayyum B., Raja N.I., Mashwani Z.U.R., Rehman S.U. (2023). Phytosynthesized nanoparticles as novel antifungal agent for sustainable agriculture: A mechanistic approach, current advances, and future directions. J. Nanotechnol..

[138] Dakal T.C., Kumar A., Majumdar R.S., Yadav V. (2016). Mechanistic basis of antimicrobial actions of silver nanoparticles. Front. Microbiol..

[139] Żarowska B., Koźlecki T., Piegza M., Jaros-Koźlecka K., Robak M. (2019). New look on antifungal activity of silver nanoparticles (AgNPs). Pol. J. Microbiol..

[140] Jozala A.F., de Lencastre-Novaes L.C., Lopes A.M., de Carvalho Santos-Ebinuma V., Mazzola P.G., Pessoa A., Chaud M.V. (2016). Bacterial nanocellulose production and application: A 10-year overview. Appl. Microbiol. Biotechnol..

[141] Terea H., Selloum D., Rebiai A., Bouafia A., Ben Mya O. (2023). Preparation and characterization of cellulose/ZnO nanoparticles extracted from peanut shells: Effects on antibacterial and antifungal activities. Biomass Convers. Biorefin..

[142] Adamczak A., Ożarowski M., Karpiński T.M. (2020). Curcumin, a natural antimicrobial agent with strain-specific activity. Pharmaceuticals.

[143] Sohn S.I., Priya A., Balasubramaniam B., Muthuramalingam P., Sivasankar C., Selvaraj A., Valliammai A., Jothi R., Pandian S. (2021). Biomedical applications and bioavailability of curcumin—An updated overview. Pharmaceutics.

[144] Amini S.M., Getso M.I., Farahyar S., Khodavaisy S., Roudbary M., Mahabadi V.P., Mahmoudi S. (2023). Antifungal activity of green-synthesized curcumin-coated silver nanoparticles alone and in combination with fluconazole and itraconazole against *Candida* and *Aspergillus* species. Curr. Med. Mycol..

[145] Rai M., Ingle A.P., Pandit R., Paralikar P., Anasane N., Santos C.A.D. (2020). Curcumin and curcumin-loaded nanoparticles: Antipathogenic and antiparasitic activities. Expert Rev. Anti Infect. Ther..

[146] Chopra H., Dey P.S., Das D., Bhattacharya T., Shah M., Mubin S., Maishu S.P., Akter R., Rahman M.H., Karthika C. (2021). Curcumin nanoparticles as promising therapeutic agents for drug targets. Molecules.

[147] Ma Z., Liu X., Nie J., Zhao H., Li W. (2022). Nano-antimicrobial peptides based on constitutional isomerism-dictated self-assembly. Biomacromolecules.

[148] Tatli Seven P., Seven I., Gul Baykalir B., Iflazoglu Mutlu S., Salem A.Z. (2018). Nanotechnology and nano-propolis in animal production and health: An overview. Ital. J. Anim. Sci..

[149] Ing L.Y., Zin N.M., Sarwar A., Katas H. (2012). Antifungal activity of chitosan nanoparticles and correlation with their physical properties. Int. J. Biomater..

[150] Al Aboody M.S., Mickymaray S. (2020). Anti-fungal efficacy and mechanisms of flavonoids. Antibiotics.

[151] Yang B., Dong Y., Wang F., Zhang Y. (2020). Nanoformulations to enhance the bioavailability and physiological functions of polyphenols. Molecules.

[152] Simonetti G., Brasili E., Pasqua G. (2020). Antifungal activity of phenolic and polyphenolic compounds from different matrices of *Vitis vinifera* L. against human pathogens. Molecules.

[153] Teodoro G.R., Ellepola K., Seneviratne C.J., Koga-Ito C.Y. (2015). Potential use of phenolic acids as anti-*Candida* agents: A review. Front. Microbiol..

[154] Nguyen T.L.A., Bhattacharya D. (2022). Antimicrobial activity of quercetin: An approach to its mechanistic principle. Molecules.

[155] Janeczko M., Gmur D., Kochanowicz E., Górka K., Skrzypek T. (2022). Inhibitory effect of a combination of baicalein and quercetin flavonoids against *Candida albicans* strains isolated from the female reproductive system. Fungal Biol..

[156] Sadeghi-Ghadi Z., Vaezi A., Ahangarkani F., Ilkit M., Ebrahimnejad P., Badali H. (2020). Potent in vitro activity of curcumin and quercetin co-encapsulated in nanovesicles without hyaluronan against *Aspergillus* and *Candida* isolates. J. Mycol. Med..

[157] Sardi J.D.C.O., Gullo F.P., Freires I.A., de Souza Pitangui N., Segalla M.P., Fusco-Almeida A.M., Rosalen P.L., Regasini L.O., Mendes-Giannini M.J.S. (2016). Synthesis, antifungal activity of caffeic acid derivative esters, and their synergism with fluconazole and nystatin against *Candida* spp.. Diagn. Microbiol. Infect. Dis..

[158] Alfarrayeh I., Pollák E., Czéh Á., Vida A., Das S., Papp G. (2021). Antifungal and anti-biofilm effects of caffeic acid phenethyl ester on different *Candida* species. Antibiotics.

[159] Yan H., Meng X., Lin X., Duan N., Wang Z., Wu S. (2023). Antifungal activity and inhibitory mechanisms of ferulic acid against the growth of *Fusarium graminearum*. Food Biosci..

[160] Zhu C., Lei M., Andargie M., Zeng J., Li J. (2019). Antifungal activity and mechanism of action of tannic acid against *Penicillium digitatum*. Physiol. Mol. Plant Pathol..

[161] Kulik T., Buśko M., Pszczółkowska A., Perkowski J., Okorski A. (2014). Plant lignans inhibit growth and trichothecene biosynthesis in *Fusarium graminearum*. Lett. Appl. Microbiol..

[162] Vo T.V., Tran N.T., Nguyen P.L., Nguyen N.N., Nguyen N.T., Nguyen T.T., Tran T.T., Nguyen V.P., Thai H.T., Hoang D. (2023). Sustainable lignin-based nano hybrid biomaterials with high-performance antifungal activity. ACS Omega.

[163] Divyashree S., Shruthi B., Vanitha P.R., Sreenivasa M.Y. (2023). Probiotics and their postbiotics for the control of opportunistic fungal pathogens: A review. Biotechnol. Rep..

[164] Shenoy A., Gottlieb A. (2019). Probiotics for oral and vulvovaginal candidiasis: A review. Dermatol. Ther..

[165] Allonsius C.N., van den Broek M.F., De Boeck I., Kiekens S., Oerlemans E.F., Kiekens F., Foubert K., Vandenheuvel D., Cos P., Delputte P. (2017). Interplay between *Lactobacillus rhamnosus* GG and *Candida* and the involvement of exopolysaccharides. Microb. Biotechnol..

[166] Poon Y., Hui M. (2023). Inhibitory effect of lactobacilli supernatants on biofilm and filamentation of *Candida albicans*, *Candida tropicalis*, and *Candida parapsilosis*. Front. Microbiol..

[167] Arasu V.M., Jung M.W., Ilavenil S., Jane M., Kim D.H., Lee K.D., Park H.S., Hur T.Y., Choi G.J., Lim Y.C. (2013). Isolation and characterization of antifungal compound from *Lactobacillus plantarum* KCC-10 from forage silage with potential beneficial properties. J. Appl. Microbiol..

[168] Ricci L., Mackie J., Donachie G.E., Chapuis A., Mezerová K., Lenardon M.D., Brown A.J., Duncan S.H., Walker A.W. (2022). Human gut bifidobacteria inhibit the growth of the opportunistic fungal pathogen *Candida albicans*. FEMS Microbiol. Ecol..

[169] Ishikawa K.H., Mayer M.P., Miyazima T.Y., Matsubara V.H., Silva E.G., Paula C.R., Campos T.T., Nakamae A.E. (2015). A multispecies probiotic reduces oral *Candida* colonization in denture wearers. J. Prosthodont..

[170] Yaragalla S., Bhavitha K.B., Athanassiou A. (2021). A review on graphene-based materials and their antimicrobial properties. Coatings.

[171] Dasari Shareena T.P., McShan D., Dasmahapatra A.K., Tchounwou P.B. (2018). A review on graphene-based nanomaterials in biomedical applications and risks in environment and health. Nano-Micro Lett..

[172] Li C., Wang X., Chen F., Zhang C., Zhi X., Wang K., Cui D. (2013). The antifungal activity of graphene oxide–silver nanocomposites. Biomaterials.

[173] Nguyen H.N., Chaves-Lopez C., Oliveira R.C., Paparella A., Rodrigues D.F. (2019). Cellular and metabolic approaches to investigate the effects of graphene and graphene oxide in the fungi *Aspergillus flavus* and *Aspergillus niger*. Carbon.

[174] Farzanegan A., Roudbary M., Falahati M., Khoobi M., Gholibegloo E., Farahyar S., Karimi P., Khanmohammadi M. (2018). Synthesis, characterization and antifungal activity of a novel formulated nanocomposite containing Indolicidin and Graphene oxide against disseminated candidiasis. J. Mycol. Med..

[175] Chen J., Peng H., Wang X., Shao F., Yuan Z., Han H. (2014). Graphene oxide exhibits broad-spectrum antimicrobial activity against bacterial phytopathogens and fungal conidia by intertwining and membrane perturbation. Nanoscale.

[176] Diez-Orejas R., Feito M.J., Cicuéndez M., Casarrubios L., Rojo J.M., Portolés M.T. (2018). Graphene oxide nanosheets increase *Candida albicans* killing by pro-inflammatory and reparative peritoneal macrophages. Colloids Surf. B Biointerfaces.

[177] Sawangphruk M., Srimuk P., Chiochan P., Sangsri T., Siwayaprahm P. (2012). Synthesis and antifungal activity of reduced graphene oxide nanosheets. Carbon.

[178] Gurunathan S., Han J.W., Dayem A.A., Eppakayala V., Park M.R., Kim J.H. (2012). Antibacterial activity of dithiothreitol reduced graphene oxide. J. Ind. Eng. Chem..

[179] Gosheger G., Hardes J., Ahrens H., Streitburger A., Buerger H., Erren M., Gunsel A., Kemper F.H., Winkelmann W., Von Eiff C. (2004). Silver-coated megaendoprostheses in a rabbit model—An analysis of the infection rate and toxicological side effects. Biomaterials.

[180] Sondi I., Salopek-Sondi B. (2004). Silver nanoparticles as antimicrobial agent: A case study on *E. coli* as a model for Gram-negative bacteria. J. Colloid Interface Sci..

[181] Alimardani V., Abolmaali S.S., Borandeh S. (2019). Antifungal and antibacterial properties of graphene-based nanomaterials: A mini-review. J. Nanostruct..

[182] Cacaci M., Martini C., Guarino C., Torelli R., Bugli F., Sanguinetti M. (2020). Graphene oxide coatings as tools to prevent microbial biofilm formation on medical devices. Adv. Microbiol. Infect. Dis. Public Health.

[183] Palmieri V., Bugli F., Cacaci M., Perini G., Maio F.D., Delogu G., Torelli R., Conti C., Sanguinetti M., Spirito M.D. (2018). Graphene oxide coatings prevent *Candida albicans* biofilm formation with a controlled release of curcumin-loaded nanocomposites. Nanomedicine.

[184] Nazzaro F., Fratianni F., Coppola R., De Feo V. (2017). Essential oils and antifungal activity. Pharmaceuticals.

[185] Mondello F., De Bernardis F., Girolamo A., Cassone A., Salvatore G. (2006). In vivo activity of terpinen-4-ol, the main bioactive component of *Melaleuca alternifolia* Cheel (tea tree) oil against azole-susceptible and-resistant human pathogenic *Candida* species. BMC Infect. Dis..

[186] Leyva-López N., Gutiérrez-Grijalva E.P., Vazquez-Olivo G., Heredia J.B. (2017). Essential oils of oregano: Biological activity beyond their antimicrobial properties. Molecules.

[187] Loc N.H., Huy N.D., Quang H.T., Lan T.T., Thu Ha T.T. (2020). Characterisation and antifungal activity of extracellular chitinase from a biocontrol fungus, *Trichoderma asperellum* PQ34. Mycology.

[188] Qingzhi W., Zou S., Wang Q., Chen L., Yan X., Gao L. (2021). Catalytic defense against fungal pathogens using nanozymes. Nanotechnol. Rev..

[189] Hartl L., Zach S., Seidl-Seiboth V. (2012). Fungal chitinases: Diversity, mechanistic properties and biotechnological potential. Appl. Microbiol. Biotechnol..

[190] Rathore A.S., Gupta R.D. (2015). Chitinases from bacteria to human: Properties, applications, and future perspectives. Enzym. Res..

[191] Ferraboschi P., Ciceri S., Grisenti P. (2021). Applications of lysozyme, an innate immune defense factor, as an alternative antibiotic. Antibiotics.

[192] Ibrahim H.R., Imazato K., Ono H. (2011). Human lysozyme possesses novel antimicrobial peptides within its N-terminal domain that target bacterial respiration. J. Agric. Food Chem..

[193] Sowa-Jasiłek A., Zdybicka-Barabas A., Stączek S., Wydrych J., Skrzypiec K., Mak P., Deryło K., Tchórzewski M., Cytryńska M. (2016). *Galleria mellonella* lysozyme induces apoptotic changes in *Candida albicans* cells. Microbiol. Res..

[194] Sebaa S., Hizette N., Boucherit-Otmani Z., Courtois P. (2017). Dose-dependent effect of lysozyme upon *Candida albicans* biofilm. Mol. Med. Rep..

[195] Nsairat H., Khater D., Sayed U., Odeh F., Al Bawab A., Alshaer W. (2022). Liposomes: Structure, composition, types, and clinical applications. Heliyon.

[196] He Y., Zhang W., Xiao Q., Fan L., Huang D., Chen W., He W. (2022). Liposomes and liposome-like nanoparticles: From anti-fungal infection to the COVID-19 pandemic treatment. Asian J. Pharm. Sci..

[197] Faustino C., Pinheiro L. (2020). Lipid systems for the delivery of amphotericin B in antifungal therapy. Pharmaceutics.

[198] Hossain R., Quispe C., Khan R.A., Saikat A.S.M., Ray P., Ongalbek D., Yeskaliyeva B., Jain D., Smeriglio A., Trombetta D. (2022). Propolis: An update on its chemistry and pharmacological applications. Chin. Med..

[199] Bouchelaghem S. (2022). Propolis characterization and antimicrobial activities against *Staphylococcus aureus* and *Candida albicans*: A review. Saudi J. Biol. Sci..

[200] Vepari C., Kaplan D.L. (2007). Silk as a biomaterial. Prog. Polym. Sci..

[201] Xue B., Zhang Y., Xu M., Wang C., Huang J., Zhang H., Zhao Q., Zhang L., Yu D., Wei Q. (2019). Curcumin-silk fibroin nanoparticles for enhanced anti-*Candida albicans* activity in vitro and in vivo. J. Biomed. Nanotechnol..

[202] Roy T.C., Ansary R.H., Biswas P., Haider M.A. (2023). Silk fibroin hydrogel-assisted controlled release of antifungal drug ketoconazole. J. Drug Deliv. Ther..

[203] Ul Haq I., Maryam S., Shyntum D.Y., Khan T.A., Li F. (2024). Exploring the frontiers of therapeutic breadth of antifungal peptides: A new avenue in antifungal drugs. J. Ind. Microbiol. Biotechnol..

[204] Böttger R., Hoffmann R., Knappe D. (2017). Differential stability of therapeutic peptides with different proteolytic cleavage sites in blood, plasma and serum. PLoS ONE.

[205] Konakbayeva D., Karlsson A.J. (2023). Strategies and opportunities for engineering antifungal peptides for therapeutic applications. Curr. Opin. Biotechnol..

[206] Yeung A.T.Y., Gellatly S.L., Hancock R.E.W. (2011). Multifunctional cationic host defence peptides and their clinical applications. Cell. Mol. Life Sci..

[207] Drayton M., Kizhakkedathu J.N., Straus S.K. (2020). Towards robust delivery of antimicrobial peptides to combat bacterial resistance. Molecules.

[208] Martin V., Egelund P.H., Johansson H., Le Quement S.T., Wojcik F., Pedersen D.S. (2020). Greening the synthesis of peptide therapeutics: An industrial perspective. RSC Adv..

[209] Raus R.A., Nawawi W.M.F.W., Nasaruddin R.R. (2021). Alginate and alginate composites for biomedical applications. Asian J. Pharm. Sci..

[210] Kong H.J., Kaigler D., Kim K., Mooney D.J. (2004). Controlling rigidity and degradation of alginate hydrogels via molecular weight distribution. Biomacromolecules.

[211] Lee K.Y., Mooney D.J. (2012). Alginate: Properties and biomedical applications. Prog. Polym. Sci..

[212] Raina N., Pahwa R., Bhattacharya J., Paul A.K., Nissapatorn V., de Lourdes Pereira M., Oliveira S.M., Dolma K.G., Rahmatullah M., Wilairatana P. (2022). Drug delivery strategies and biomedical significance of hydrogels: Translational considerations. Pharmaceutics.

[213] Salthouse D., Novakovic K., Hilkens C.M., Ferreira A.M. (2023). Interplay between biomaterials and the immune system: Challenges and opportunities in regenerative medicine. Acta Biomater..

[214] Flemming H.C., Wingender J., Szewzyk U., Steinberg P., Rice S.A., Kjelleberg S. (2016). Biofilms: An emergent form of bacterial life. Nat. Rev. Microbiol..

[215] Teng Y., Li S., Tang H., Tao X., Fan Y., Huang Y. (2023). Medical applications of hydrogels in skin infections: A review. Infect. Drug Resist..

[216] Kravanja G., Primožič M., Knez Ž., Leitgeb M. (2019). Chitosan-based (Nano) materials for novel biomedical applications. Molecules.

[217] Aranaz I., Alcántara A.R., Civera M.C., Arias C., Elorza B., Heras Caballero A., Acosta N. (2021). Chitosan: An overview of its properties and applications. Polymers.

[218] Shrestha R., Thenissery A., Khupse R., Rajashekara G. (2023). Strategies for the preparation of chitosan derivatives for antimicrobial, drug delivery, and agricultural applications: A review. Molecules.

[219] Zoe L.H., David S.R., Rajabalaya R. (2023). Chitosan nanoparticle toxicity: A comprehensive literature review of in vivo and in vitro assessments for medical applications. Toxicol. Rep..

[220] Rai M., Yadav A., Gade A. (2009). Silver nanoparticles as a new generation of antimicrobials. Biotechnol. Adv..

[221] Rasmussen J.W., Martinez E., Louka P., Wingett D.G. (2010). Zinc oxide nanoparticles for selective destruction of tumor cells and potential for drug delivery applications. Expert Opin. Drug Deliv..

[222] Maheswaran H., Djearamane S., Dhanapal A.C.T.A., Shing W.L. (2024). Cytotoxicity of green synthesized zinc oxide nanoparticles using *Musa acuminata* on vero cells. Heliyon.

[223] Khlebtsov N., Dykman L. (2011). Biodistribution and toxicity of engineered gold nanoparticles: A review of in vitro and in vivo studies. Chem. Soc. Rev..

[224] Derfus A.M., Chan W.C., Bhatia S.N. (2004). Probing the cytotoxicity of semiconductor quantum dots. Nano Lett..

[225] Navarro E., Piccapietra F., Wagner B., Marconi F., Kaegi R., Odzak N., Sigg L. (2008). Toxicity of silver nanoparticles to Chlamydomonas reinhardtii. Environ. Sci. Technol..

[226] Ganesan K., Xu B. (2017). Polyphenol-rich dry common beans (*Phaseolus vulgaris* L.) and their health benefits. Int. J. Mol. Sci..

[227] Anand P., Kunnumakkara A.B., Newman R.A., Aggarwal B.B. (2007). Bioavailability of curcumin: Problems and promises. Mol. Pharm..

[228] Weber K., Schulz B., Ruhnke M. (2011). Resveratrol and its antifungal activity against *Candida* species. Mycoses.

[229] James A., Wang K., Wang Y. (2023). Therapeutic activity of green tea epigallocatechin-3-gallate on metabolic diseases and non-alcoholic fatty liver diseases: The current updates. Nutrients.

[230] Daglia M. (2012). Polyphenols as antimicrobial agents. Curr. Opin. Biotechnol..

[231] Li Z., Chen Z., Chen H., Chen K., Tao W., Ouyang X.K., Mei L., Zeng X. (2022). Polyphenol-based hydrogels: Pyramid evolution from crosslinked structures to biomedical applications and the reverse design. Bioact. Mater..

[232] Sanchez V.C., Jachak A., Hurt R.H., Kane A.B. (2012). Biological interactions of graphene-family nanomaterials: An interdisciplinary review. Chem. Res. Toxicol..

[233] Chang Y., Yang S.T., Liu J.H., Dong E., Wang Y., Cao A., Liu Y. (2011). In vitro toxicity evaluation of graphene oxide on A549 cells. Toxicol. Lett..

[234] Sontakke A.D., Tiwari S., Purkait M.K. (2023). A comprehensive review on graphene oxide-based nanocarriers: Synthesis, functionalization and biomedical applications. FlatChem.

[235] Kumar P., Huo P., Zhang R., Liu B. (2019). Antibacterial properties of graphene-based nanomaterials. Nanomaterials.

[236] Yusaf T., Mahamude A.S.F., Farhana K., Harun W.S.W., Kadirgama K., Ramasamy D., Kamarulzaman M.K., Subramonian S., Hall S., Dhahad H.A. (2022). A comprehensive review on graphene nanoparticles: Preparation, properties, and applications. Sustainability.

[237] Rose Jørgensen M., Thestrup Rikvold P., Lichtenberg M., Østrup Jensen P., Kragelund C., Twetman S. (2020). *Lactobacillus rhamnosus* strains of oral and vaginal origin show strong antifungal activity in vitro. J. Oral Microbiol..

[238] Han S., Lu Y., Xie J., Fei Y., Zheng G., Wang Z., Liu J., Lv L., Ling Z., Berglund B. (2021). Probiotic gastrointestinal transit and colonization after oral administration: A long journey. Front. Cell. Infect. Microbiol..

[239] Hill C., Guarner F., Reid G., Gibson G.R., Merenstein D.J., Pot B., Morelli L., Canani R.B., Flint H.J., Salminen S. (2014). The international scientific association for probiotics and prebiotics consensus statement on the scope and appropriate use of the term probiotic. Nat. Rev. Gastroenterol. Hepatol..

[240] Martinez R.C.R., Franceschini S.A., Patta M.C., Quintana S.M., Candido R.C., Ferreira J.C., De Martinis E.C.P., Reid G. (2009). Improved treatment of vulvovaginal candidiasis with fluconazole plus probiotic Lactobacillus rhamnosus GR-1 and Lactobacillus reuteri RC-14. Lett. Appl. Microbiol..

[241] Petrariu O.A., Barbu I.C., Niculescu A.G., Constantin M., Grigore G.A., Cristian R.E., Mihaescu G., Vrancianu C.O. (2024). Role of probiotics in managing various human diseases, from oral pathology to cancer and gastrointestinal diseases. Front. Microbiol..

[242] Mikucka A., Deptuła A., Bogiel T., Chmielarczyk A., Nurczyńska E., Gospodarek-Komkowska E. (2022). Bacteraemia caused by probiotic strains of *Lacticaseibacillus rhamnosus*—Case studies highlighting the need for careful thought before using microbes for health benefits. Pathogens.

[243] Suez J., Zmora N., Zilberman-Schapira G., Mor U., Dori-Bachash M., Bashiardes S., Zur M., Regev-Lehavi D., Brik R.B.Z., Federici S. (2018). Post-antibiotic gut mucosal microbiome reconstitution is impaired by probiotics and improved by autologous FMT. Cell.

[244] Goy R.C., Britto D.D., Assis O.B.G. (2016). A review of the antimicrobial activity of chitosan. Polímeros.

[245] Assoni L., Milani B., Carvalho M.R., Nepomuceno L.N., Waz N.T., Guerra M.E.S., Converso T.R., Darrieux M. (2020). Resistance mechanisms to antimicrobial peptides in gram-positive bacteria. Front. Microbiol..

[246] Yaraghi N.A., Kisailus D. (2018). Biomimetic structural materials: Inspiration from design and assembly. Annu. Rev. Phys. Chem..

[247] Pereira P.M.M., Monteiro G.A., Prazeres D.M.F. (2015). General aspects of biomimetic materials. Biotechnologies and Biomimetics for Civil Engineering.

[248] Khalifa H.O., Oreiby A.F., Abd El-Hafeez A.A., Okanda T., Haque A., Anwar K.S., Tanaka M., Miyako K., Tsuji S., Kato Y. (2020). First report of multidrug-resistant carbapenemase-producing bacteria coharboring *mcr-9* associated with respiratory disease complex in pets: Potential of animal-human transmission. Antimicrob. Agents Chemother..

[249] Khalifa H.O., Oreiby A., Abd El-Hafeez A.A., Abd El Latif A., Okanda T., Kato Y., Matsumoto T. (2021). High β-lactam and quinolone resistance of *Enterobacteriaceae* from the respiratory tract of sheep and goat with respiratory disease. Animals.

[250] Khalifa H.O., Oreiby A.F., Okanda T., Kato Y., Matsumoto T. (2021). High β-lactam resistance in Gram-negative bacteria associated with kennel cough and cat flu in Egypt. Sci. Rep..

[251] Khalifa H.O., Shikoray L., Mohamed M.-Y.I., Habib I., Matsumoto T. (2024). Veterinary drug residues in the food chain as an emerging public health threat: Sources, analytical methods, health impacts, and preventive measures. Foods.

[252] Lu Q., Okanda T., Yang Y., Khalifa H.O., Haque A., Takemura H., Matsumoto T. (2021). High-speed quenching probe-polymerase chain reaction assay for the rapid detection of carbapenemase-producing gene using GENECUBE: A fully automatic gene analyzer. Mol. Diagn. Ther..

[253] Habib I., Elbediwi M., Mohteshamuddin K., Mohamed M.Y.I., Lakshmi G.B., Abdalla A., Anes F., Ghazawi A., Khan M., Khalifa H. (2023). Genomic profiling of extended-spectrum β-lactamase-producing *Escherichia coli* from pets in the United Arab Emirates: Unveiling colistin resistance mediated by *mcr-1.1* and its probable transmission from chicken meat—A One Health perspective. J. Infect. Public Health.

[254] Khalifa H.O., Al Ramahi Y.M. (2024). After the Hurricane: Anti-COVID-19 drugs development, molecular mechanisms of action and future perspectives. Int. J. Mol. Sci..

